# Uncoupling growth and division in *Chlamydomonas reinhardtii* colonies: consistent cell cycle regulation under confinement

**DOI:** 10.1093/ismeco/ycaf104

**Published:** 2025-06-23

**Authors:** Sing Teng Chua, Jurij Kotar, Michael Kühl, Alison G Smith, Silvia Vignolini, Pietro Cicuta

**Affiliations:** Yusuf Hamied Department of Chemistry, University of Cambridge, Lensfield Road, Cambridge CB2 1EW, United Kingdom; Cavendish Laboratory, University of Cambridge, JJ Thomson Avenue, Cambridge CB3 0US, United Kingdom; Marine Biology Section, Department of Biology, University of Copenhagen, Strandpromenaden 5, Helsingør, DK-3000, Denmark; Department of Plant Sciences, University of Cambridge, Downing Street, Cambridge, CB2 3EA, United Kingdom; Yusuf Hamied Department of Chemistry, University of Cambridge, Lensfield Road, Cambridge CB2 1EW, United Kingdom; Department of Sustainable and Bio-inspired Materials, Max Planck Institute of Colloids and Interfaces, Am Mühlenberg 1 OT Golm, 14476 Potsdam, Germany; Cavendish Laboratory, University of Cambridge, JJ Thomson Avenue, Cambridge CB3 0US, United Kingdom

**Keywords:** cell cycle, confined growth, cell size regulation

## Abstract

A planar cell microcolony served as a model system to study the impact of inter-cellular crowding and cell-matrix interactions upon the cell cycle. We studied the development over several days of *Chlamydomonas reinhardtii* microcolonies, grown from single cells, using a bespoke experimental setup allowing timelapse fluorescence microscopy. Through precise cell segmentation and lineage tracking of a large systematic dataset, characterising individual cell growth and divisions, we uncovered how the external matrix influenced cell cycle and morphology. Experiments also revealed spatial heterogeneity amongst cells within colonies, providing insights into the effects of contact inhibition and micro-gradients of mass transfer. A radial propagation of ring-like pattern, characterised by variations in parent cell size, indicated complex spatio-temporal dynamics in the regulation of the cell cycle within the constrained environment. The mechanisms of commitment and mitotic sizing remained consistent within colonies under this mechanical confinement. These findings contribute to a broader understanding of how matrix immobilisation affects *C. reinhardtii*, with implications for alternative culture formats such as biofilms and hydrogel encapsulation—approaches increasingly used in biohybrid applications including biophotovoltaics and bioremediation.

## Introduction

The cell cycle in microalgae such as *Chlamydomonas reinhardtii* begins with cellular size enlargement of up to 10-fold known in the growth phase (G1), followed by successive rounds of rapid cell divisions to produce 2^n^ daughter cells [1], where DNA replication (S) takes place followed by mitosis (nucleus division) and cytokinesis (cytoplasm division), denoted as M. Synchronisation can be achieved by diurnal light/dark cycles, where G1 occurs during illumination while S/M divisions take place in the dark [2]. At the end of the cell cycle, daughter cells hatch out of the mother cell and restart the cycle from G1. Typically, G1 can last between 10 and 14 h while each S/M spans ~30–40 min [3]. The cell cycle is known to depend on a large number of factors, including temperature [4], light intensity [,5], and nutrient supply [6].

The regulation of this multiple fission process is through the role of control points, namely ‘sizer’ and ‘timer’. In *C. reinhardtii*, the cell cycle features a cut-off size threshold, known as the primary arrest point in G1 phase, beyond which cells become fully committed to completing the cell cycle, with or without growth, independently of external conditions, such as light availability [7]. A mitotic sizer at S/M phase correlates the number of daughter cells 2^n^ with the size of the mother cell at the end of G1 phase, considering equal and uniform size distribution across all daughter cells [8, 9]. The number of divisions *n* relies upon the growth condition but falls between 1 and 5. During the precommitment phase of growth, cells increase in size with the fabrication of cellular organelles and the accumulation of energy reserves. In the postcommitment phase, light-independent processes such as cell division could be observed at the end of the light phase, taking place over 4 h into the dark phase, utilizing internal energy reserves collected in the former stage. Cell division in *C. reinhardtii* involves an iterative sequence of DNA replication (S phase), nuclear division (mitosis), and cytokinesis, typically occurring in multiple alternating rounds. For example, during multiple fission, three successive cycles of S–M–C may be executed to produce up to eight daughter cells [1].

Conventionally, studies focus on the population-averaged properties of a bulk culture containing up to thousands of millions of cells, be it liquid suspension, surface colonies or encapsulated culture. The statistical mean reflects the representative behaviour of a phenotype that is unique for a typical genotype in a specific environmental condition. All these approaches are based on an important assumption of homogeneity in sampling population and cellular behaviours, which often does not hold true upon scrutiny at single cell [10]. External perturbations in the surrounding environment contribute further to cell–cell heterogeneity [11], including micro-environmental differences, varied species within microbial consortia, stochastic gene expression, etc. [12]. Hence, single-cell analysis is a key to essential information much needed for accurate interpretation and understanding of heterogeneity and its significance in regulating cellular processes.

In the past decades, microfluidic systems have been developed for high-throughput single-cell studies of microalgae with precise spatio-temporal and culture control such as illumination conditions, temperature, carbon dioxide concentration, and nutrient composition [13]. Using these platforms, it is possible to study dynamic cell–cell interactions [14], cell migration [15], strain screening [16], growth kinetics [17], photosynthetic activity [18], etc. However, the investigation of cell growth kinetics and detailed cell morphology still requires live-cell microscopy imaging [19]. With the exception of Lee *et al.* who utilised alginate hydrogel microcapsules to study the heterogeneity in lipid content amongst single cells of *Chlorella vulgaris*, *Chlamydomonas sp* and *Botryococcus braunii* [20], almost all single-cell studies focus on liquid suspension in the form of microfluidic flow or microdroplets [21]. Surface immobilisation with microfluidics was also attempted for continuous monitoring of lipid droplet accumulation in *C. reinhardtii* [19]. Utilizing a microfluidic device, Min *et al.* [22] revealed an extended G1 duration under mechanical stress. Nevertheless, a detailed investigation of cell cycle behaviour upon immobilisation is still lacking in *C. reinhardtii*, and the growth kinetics and the mechanism of cell colony formation are yet unknown. No prior studies have investigated the effect of contact inhibition on the multiple fission of *C. reinhardtii*.

One key objective of this work aims at studying the impact of matrix immobilisation on *C. reinhardtii*, providing biological insights into less conventional methods of algal culture such as biofilm attachment [23, 24] and hydrogel encapsulation [25, 26]. Understanding the biological and physiological effects of cell confinement provides crucial insights into the early stages of biofilm formation in *C. reinhardtii*. While existing research on bio-composite materials frequently focuses on material properties and functional aspects, there remains a significant gap in the comprehension of cellular behaviour following gel immobilisation. A detailed understanding of cell–cell heterogeneity and spatial distribution is essential, as it can inform the design of future applications requiring spatial precision or specific correlations in engineered living materials.

Here, we build on experimental and conceptual frameworks we developed recently for bacteria [27, 28], utilizing agarose gel pads to limit the growth of cell colonies in two dimensions, and developing a pipeline of image analysis to follow the individual cells within isolated colonies. Thanks to a customised setup that supports stable long-term time-lapse microscopy, allowing many courses of cell cycle to be captured under varying conditions, we reveal patterns of spatiotemporal heterogeneity in single-cell growth and division within colonies, and show that cell division sizer control remains robust even in confinement.

## Materials and methods

### Cell culture

*C. reinhardtii* wild-type strain 12 (WT-12), derived from strain CC-124 (137c mt^-^ nit1 nit2), was used in this study. The parental strain CC-124 is available from the Chlamydomonas Resource Centre (https://www.chlamycollection.org/product/cc-124-wild-type-mt-137c/). *C. reinhardtii* was maintained in organic carbon supplemented Tris-acetate-phosphate (TAP) medium (Tris base: 20.043 mM; Beijernick salts (NH_4_Cl: 7.477 mM, MgSO_4_·7H_2_O: 406 μM, and CaCl_2_·2H_2_O: 340 μM); phosphate solution (K_2_HPO_4_: 620 μM, KH_2_PO_4_: 412 μM); Kropat’s trace elements (EDTA-Na_2_: 57.8 μM, (NH_4_)_6_Mo_7_O_24_: 0.0285 μM, and ZnSO_4_·7H_2_O: 2.5 μM; MnCl_2_·4H_2_O: 50 mg L^−1^; FeCl_3_·6H_2_O: 20 μM; CuCl_2_·2H_2_O: 2 μM); CoCl_2_: 7.7 μM; glacial acetic acid: 17.416 mM) [29, 30]. Prior to imaging, the planktonic culture was grown for 5 days in an orbital incubator (Infors HT Multitron Pro.) at 25°C with shaking at 100 rpm under a diurnal cycle of 12 h light (100 μmol photons m^−2^·s^−1^) and 12 h dark. In addition to TAP medium, a Tris-minimal medium was also used in selected experiments. Tris-minimal medium is compositionally similar to TAP, except for the absence of glacial acetic acid, making it suitable for photoautotrophic rather than mixotrophic growth.

### Gel immobilisation for imaging

To obtain the seed cultures for imaging purpose, the precultures were first diluted to an optical density at 750 nm of 0.1 and then further diluted by 100 times by 10-fold serial dilution. The cell density was kept sufficiently low to ensure that cells were sparse and well separated at the onset of the experiment. This setup guaranteed that under the imaging frame at the beginning of the time-lapse monitoring, individual cells appeared isolated. To immobilise *C. reinhardtii* and ensure the formation of planar microcolonies, 20 μL of this seed culture was sandwiched between agarose gel pad containing TAP nutrient medium and a gas-permeable polymer coverslip that made up the bottom observation area (20 mm in diameter) of a 35 mm culture dish (Ibidi GmbH, Gräfelfing, Germany). This setup confined the cells within a coverslip-gel interface, facilitating their division and growth in a planar orientation (Fig. S1). In this work, the concentration of agarose gel pads varied between 0.5% weight/weight (% wt) and 3% wt of agarose (Sigma-Aldrich, A9539) in TAP medium.

In rare cases, cells within the microcolony exhibited transient swimming motion due to local lifting or detachment of the agarose gel from the substrate. These occurrences were identified as artefacts, as mechanical confinement from the gel matrix and cell crowding otherwise effectively suppressed motility. Such frames were excluded from downstream analysis to ensure that only sessile, matrix-associated cells were analysed.

### Timelapse microscopy with environmental control

Each experimental run in this work typically involved continuous epifluorescence timelapse imaging. To optimise this process, a bespoke open-frame automated microscope was designed and constructed (Fig. S2). The data in this paper corresponded to continuous imaging for up to a week. Longer tracking beyond 2 weeks was also feasible with this setup. All motorised functions of the microscope (stage movement, autofocus, turret rotation of filter sets for fluorescence) and all illumination properties (day/night, transmission and epifluorescence lights) as well as camera image acquisition were controlled by an in-house software platform called Temika, written in C.

The microscope was equipped with a detachable stage accommodating eight individual culture dishes (Ibidi μ-Dish 35 mm, 81 151). Dishes were imaged sequentially, depending on the number and distribution of microcolonies (typically 20–40 colonies per dish). The imaging cycle was repeated every hour, with the 1-h interval measured from the acquisition time of the first image in each cycle. While the stage was screwed to the microscope chassis to minimise unwanted motion, the top of the stage was covered with a clear heated glass panel to prevent condensation on the top of the Ibidi dishes. The entire system was enclosed within an opaque and insulated cabinet, designed to maintain temperature stability using a Proportional–integral–derivative temperature controller. Light illumination at 80 μmol photons m^−2^·s^−1^ was provided to the algae with two strips of blue and red LEDs positioned close to the dish holder (Fig. S2B), similar to the environment of a culture incubator. A constant flow of humidified air was streamed through the imaging chamber to maintain humidity levels and prevent gel dehydration over time. An external fluid delivery system connected a syringe, operated by a pump, to the Ibidi dish through microfluidic tubing (outer diameter 3 mm; inner diameter 1 mm). The LED illumination was turned off automatically and momentarily during image capturing to ensure accurate data capture without interference from ambient light.

The microscope was built in an inverted configuration and equipped with a Nikon 40x Plan Fluor objective (working distance (WD) 0.66, numerical aperture (NA) 0.75) and an Andor Zyla 5.5 camera for imaging in brightfield transmission and epifluorescence mode. An Auto-Focus System (AFS) based on an optical lever was used to counteract axial focus fluctuations caused by undesired vibrations or thermal drift during timelapse imaging, which are inevitable over long times, especially when moving repeatedly across multiple Ibidi dishes.

As a first step, a visual scanning within a dish was performed to identify the position of single well-isolated algal cells. Once the AFS was activated, the specific x, y, z coordinates and AFS offset required for the focal plane were recorded and stored for each cell. A range of 20 to 30 positions were selected within each dish in this way. Once all desired cell positions to be tracked had been selected, the number of scans and lighting cycle setting were set. Subsequently, a command script was generated in Temika to initiate the experiment with automated stage movement and capture images at each position. The exposure time at respective channels of brightfield with green illumination, brightfield with blue illumination and fluorescence with 470 nm-excitation could be adjusted according to experimental needs. The system was outfitted with a Semrock LED-DA/FI/TR/Cy5-B-000 quad band fluorescence filter set, which is designed for imaging across four windows: 414–450 nm, 500–530 nm, 580–611 nm, and 661–800 nm. In this work, chlorophyll autofluorescence of *C. reinhardtii*, which has an emission peak at 700 nm, was primarily used for image processing and analysis. The imaging interval was adjustable and set to be 1 h. It would be possible to select a higher number of cell positions, but the trade-off would be a longer imaging interval. Care was taken to make sure that the stage returned to its origin, corresponding to the centre position amongst eight dishes between rounds of imaging for equal exposure to the illuminating LED panel across all cells studied. Cells undergoing timelapse imaging were subjected to 12-h light–dark cycle of 80 μmol photons m^−2^·s^−1^ under the red and blue LED panels, following the same diurnal cycle incubating the liquid precultures, to achieve diurnal synchronisation.

### Image segmentation and analysis

The images were analysed using a semi-customised processing pipeline that utilised a combination of multiple platforms. Initially, Fiji (version 2.14.0/1.54f) [31] was employed to crop and centre the images, compensating for the slight drift caused by the mechanical movement of the microscope stage during timelapse looping. The image cropping step was implemented to save computational cost and time in the subsequent steps of segmentation. By focusing on the regions of interest and eliminating extraneous areas, this preprocessing step streamlined the analysis process, making the segmentation and feature extraction more efficient. To ensure data quality, all time-lapse sequences were manually inspected prior to analysis. Regions of the image that were out of focus—most commonly occurring at the periphery of the growing colony—were excluded. Additionally, analysis was limited to time points before the colony expanded beyond the microscope’s field of view, in order to prevent edge-related artefacts or data loss. Following this, individual cells were segmented using Stardist [32], facilitated by a custom Python script, specifically in the chlorophyll fluorescence channel. The effectiveness of the segmentation process in distinguishing individual cells is demonstrated in a representative example (Fig. S3A). To generate the ground truth dataset used for model training in Stardist and for evaluating segmentation accuracy, cells were first segmented using a semi-automated approach with Cellpose [33]. The resulting masks were then manually reviewed and edited to correct segmentation errors, to be used as the ground truth data. The performance metrics for cell segmentation, calculated from 20 microcolonies comprising a total of 920 cells (Fig. S3B). The segmented images and their corresponding masks were then analysed using CellProfiler [34] to extract various features, including cell positions, area, fluorescence intensity, and morphological properties.

### Differentiation of growth and division phases

To differentiate between the growth (G1) and division (S/M) phases, we initially explored the use of supervised machine learning via CellProfiler Analyst, which classified cells based on extracted morphological features. This approach was used illustratively to visualise cell cycle phase separation; however, it was not applied in the main quantitative analysis due to limited classification accuracy and inconsistency across colonies and imaging conditions. The examination of cell size variation over time involved identifying peaks and valleys in the data (Fig. S4). Peaks corresponded to periods when the average cell size was at its maximum, while valleys represented periods of minimum average cell size. It is noted that the abrupt drop in cell size during division aligns with a corresponding step-like increase in cell number within the microcolony. This temporal pattern highlighted fluctuations in cell growth and division cycles, allowing separate analyses of the parent and daughter cells. This method effectively distinguished between parent and daughter cells when the two populations exhibited a clear bimodal distribution. Cell populations could display a bimodal distribution when the daughter and parent cells co-exist in the same frame, indicating two distinct populations of cell sizes within the microcolonies (Fig. S5A and B). This phenomenon, coupled with bimodal decoupling, allowed for a detailed analysis of cell size across temporal variation.

### Cell division analysis

The images were analysed by comparing consecutive frames with pairing information based on cell location and cell size, allowing for the tracking of cells as they transitioned into mitosis. In the analysis of positional correlation between consecutive frames, cells entering the mitotic phase were identified and highlighted. These cells, along with their corresponding parent and daughter groups, were colour-coded for clarity (Fig. S6). The algorithm considered cells within a narrow range of positional coordinates and filtered them based on changes in cell size between frames. Specifically, cells were tracked from one frame to the next, and those that exhibited a decrease in size were identified as likely to have undergone mitosis. To reconstruct division histories and quantify cell proliferation dynamics, we implemented a custom lineage tracking algorithm based on spatial and morphological features across sequential time-lapse frames. To initiate lineage assignment, cells in adjacent frames were matched based on spatial proximity using a KD-tree structure, with the search radius set to 1.5 times half the equivalent diameter of the parent cell. For a match to be considered a valid parent–daughter relationship, the daughter’s area was required to be at least 1.5 times smaller than the parent’s area (adjustable via a min_size_drop_ratio parameter), ensuring that only size-consistent divisions were included. To accommodate divisions that span multiple frames—particularly in the case of successive divisions within a single cell cycle—the algorithm allowed a frame gap of up to five frames (max_frame_gap = 5) before disassociating a cell from its prior identity. This helps avoid misclassification of slowly dividing cells or closely positioned sibling cells.

### Microsensor measurements of oxygen

*C. reinhardtii* colonies were grown in a 1% wt agarose gel with a very low cell inoculum density, creating sparsely distributed algal aggregates within a 35-mm glass-bottomed μ-dish. A cylindrical opaque enclosure served as the sample dish holder. The specimen was illuminated from the bottom of the observation dish using a LED light source (KL-2500, Schott GmbH, Germany) with controlled intensity. A fiber-optic scalar irradiance microprobe with a 40 μm spherical tip diameter was adopted to measure spectral scalar irradiance depth profiles from above [35]. Measurements were taken in vertical steps of 50 μm at various lateral positions as replicates across the immobilised algal aggregates [35–37]. The positioning of the probe tip was precisely controlled using a motorised micromanipulator system, operated by a custom-built software [38]. The spatial profiles of oxygen concentration were measured across isolated *C. reinhardtii* aggregates immobilised within gel matrix using a Clark-type O_2_ microsensor, featuring a tip size of 25 μm, 90% response time of <0.5 s and a stirring sensitivity of ∼1% (OX-25, Unisense A/S, Aarhus, Denmark) [39]. The sensor readings were calibrated in an air-saturated and anoxic Tris-minimal medium. Depending on the experimental temperature and salinity, the percentage of air saturation was converted to absolute oxygen concentration (μmol O_2_ L^−1^) based on the tabulated values of oxygen solubility in water (Ramsing and Gundersen, Unisense, Denmark; www.unisense.com). All oxygen microsensor measurements were conducted with high positional precision using a custom-built motorised micromanipulation system. The O₂ microsensor was mounted on a platform comprising three interconnected motorised linear stages (VT-80, Micos GmbH, Germany), controlled by MoCo DC controllers (Micos GmbH, Germany), allowing for precise positioning in the x, y, and z dimensions with a resolution of ~1 μm. Vertical profiling was performed using a step interval of 25 μm. Both positioning and data acquisition were managed by a custom software interface (Volfix) developed in LabView (National Instruments, Japan) [38]. For two-dimensional transect measurements of oxygen concentration, the O₂ microsensor tip was manually positioned as close as possible to the surface of the agarose gel using a USB microscope (Dino-Lite) for visual guidance. This position was defined as *z* = 0 in the Volfix measurement software. Laterally, the microsensor tip was initially positioned ~0.2 mm beyond the edge of the visible colony boundary. Transect measurements were initiated from this starting point and proceeded laterally across the colony, with vertical O₂ concentration profiles recorded at each step. A lateral resolution of 0.5 mm was used along the *y*-axis, and vertical profiles were collected in 0.25 mm increments along the *z*-axis. The vertical descent at each transect point continued until the tip exited the colony region, as inferred from a sudden increase in transmitted light intensity. Transect measurements were terminated once the entire colony cross-section had been scanned. A constant air stream was introduced to agitate the liquid surface gently in a circular motion, in order to minimise the buildup of a thick diffusive boundary layer that could compromise the oxygen transport across air-liquid interface.

## Results

### Light–dark synchronisation of microcolonies

**Figure 1:**
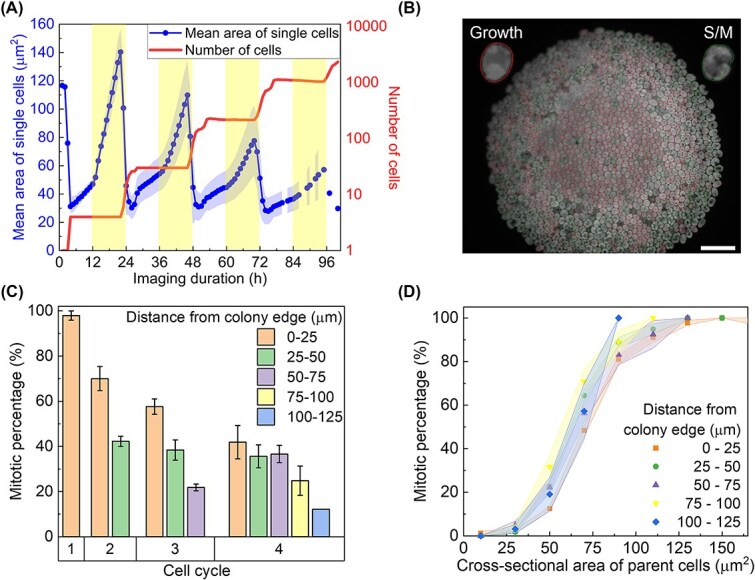
Synchronisation is lost in large colonies—radial patterns emerge. (A) Timelapse monitoring of individual microcolonies revealed periodic expansion in total cell number corresponding to diurnal illumination, driven by the growth-division synchronisation of individual cells with the light–dark cycle. The light phase is indicated by yellow shading, highlighting periods of illumination. This figure shows the mean cell area and the total cell number in a single microcolony, under 0.5% wt agarose gel pad enriched with TAP nutrient medium. The error band represents the standard error of the single cell area at each frame. (B) Illustration of cell cycle phase classification based on morphological features. Using machine learning, the segmented cells were classified into two general cell cycle phases of growth (G) with red outlines and synthesis/mitosis (S/M) with green outlines (scale bar = 50 μm). Cells in the outermost region divided earlier than those located closer to the microcolony centre. (C) Mitotic percentage across the population was computed within concentric rings positioned at varying distances from the colony edge at the time of imaging, under a 0.5% wt agarose gel pad. Error bars represent the standard error (n = 8 microcolonies). The ‘number of cycles’ refers to the number of discrete division events observed within a colony. For example, a colony exhibiting its first division at 2 h and a second division at 26 h would be considered to have undergone two division cycles. Mitotic percentage was calculated using consecutive frames selected around the onset of division timepoints. (D) The dependence of mitotic percentage on the size of cells positioned at varied radial distances from the colony edge, under a 0.5% wt agarose gel pad. The error band indicates standard error of mean (*n* = 21 microcolonies).

To provide spatial confinement while maintaining gas exchange, microcolonies were immobilised between a nutrient-enriched TAP agarose gel pad and a gas-permeable polymer coverslip (Fig. S1). This setup allowed continuous microscopic observation of colony development in a quasi-two-dimensional plane over multiple cell cycles. The microcolonies were subjected to a 12-h light/12-h dark cycle throughout the course of the timelapse imaging. The diurnal synchronisation of *C. reinhardtii* with the light–dark cycles is reflected in the cyclic pattern of division in the dark, and growth in the light period [40]. The same behaviour was captured in our system using *C. reinhardtii*, in a representative example where a single cell underwent G1 growth phase during the light period on the first day before undergoing multiple fissions to divide into four daughter cells, which then started dividing into 32 cells after another 24 h (Fig. 1A and S7). Further inspection of the single cell size revealed similar information: the cross-sectional area of a single cell increased from 30 μm^2^ up to 140 μm^2^ during the initial growth phase and decreased abruptly once cell division was initiated at the mark of 22nd hour, concurrently with the increase in cell number within the microcolony. Notably, the mean cell area increased faster in the light phase compared to the dark phase when only heterotrophic acetate consumption was possible. The abrupt decrease in mean cell size corresponded to cell division upon light–dark transition, as reflected in the step-like increase in cell number. The slight reduction in cell numbers between the 80th and 96th hours was attributed to the lateral and vertical expansion of the microcolony [41]. During the light phase, the microcolony expands as individual cells grew, yet the total number of cells remained constant due to the absence of mitotic events. Furthermore, as cells grew and compressed against neighbouring cells, some migrated into the adjacent cell layer further from the imaging plane and, constrained by the finite size of the imaging frame, cells that were pushed beyond the field of view could not be captured.

### Spatial heterogeneity of cell cycle behaviour

This subsection aims to document the variation in cell cycle progression relative to the radial positions within a microcolony, to understand how cell crowding might influence the mechanisms of commitment sizer and mitotic sizer. One primary observation was the loss of cell cycle synchronisation across the colony: whilst the peripheral cells grew and divided in accordance with the diurnal illumination, the cells in the middle displayed a phase lag to grow and divide. This loss of synchronisation was clearly observed when the cells were classified into the phases of growth (G) and synthesis/mitosis (S/M) via machine learning (Fig. 1B). The question arises whether the observed phase lag in cell cycle phases near the microcolony centre was due to a reduced division frequency, delayed division timing, or a slower cell growth rate that prevented cells from reaching the commitment threshold for division. To investigate this, we quantified the spatial distribution of actively dividing cells by calculating the mitotic percentage, defined as the fraction of cells that entered the S/M phase between the current and subsequent imaging frames. The calculation was performed within defined spatial and cell size bins, with the denominator representing the total number of cells present in the current frame within each respective bin. The mitotic percentage decreased with each successive cell cycle progression, where the effect of cell crowding had increased with the number of cells (Fig. 1C). After four cell cycles, the microcolony had expanded to reach the size of more than 100 μm in radius, accompanied by a decrease in mitotic percentage with the distance of the cell from the edge of the microcolony.

Further inspection shows that given the same size of parent cells, the mitotic percentage did not change with respect to radial distance (Fig. 1D and Figure S8). Indeed, a linear dependence between mitotic rate and parent cell size was observed below a threshold of around 75 μm^2^, a typical sizer response. Beyond this commitment threshold, there was a reduction in the gradient of mitotic rate with parent cell size, approaching a plateau as the mitotic rate saturated. Hence, the phase delay in cell division across radial distance was primarily due to slower growth rate and the longer time required to attain commitment threshold in the middle.

**Figure 2:**
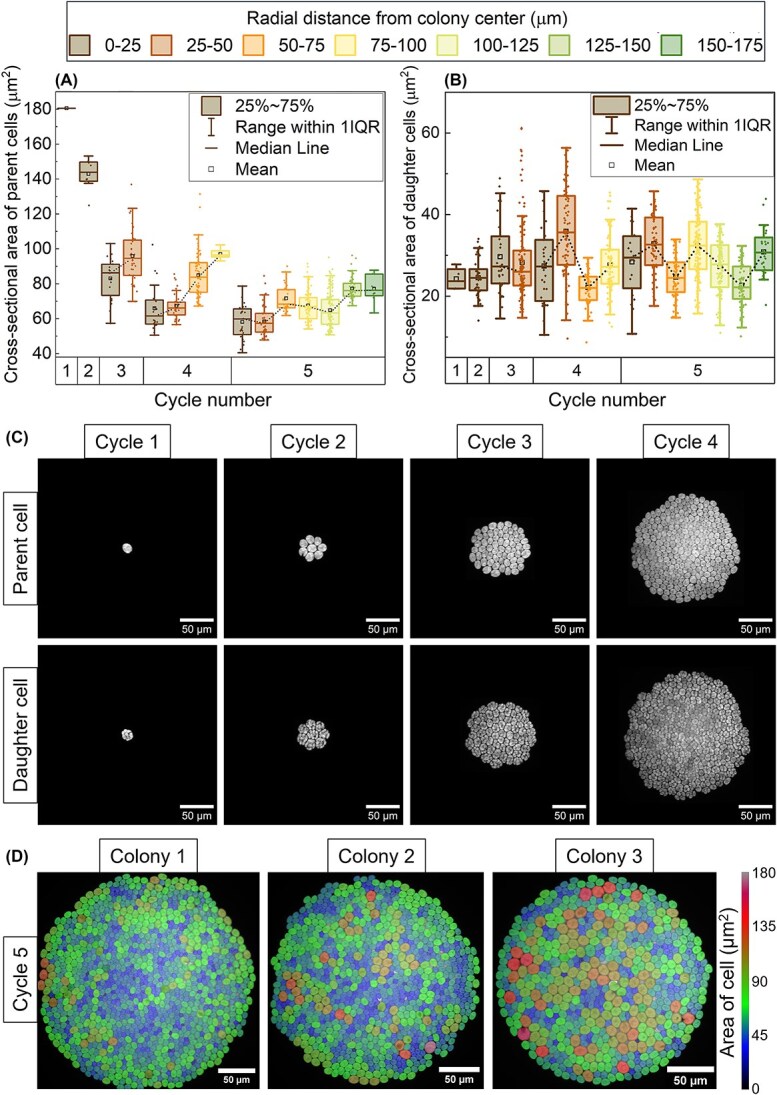
Parent–daughter cell dynamics across division cycles in microcolonies. Box and whisker plots comparing the cross-sectional area of (A) parent cells and (B) daughter cells at different radial positions upon each cycle of divisions within a single microcolony under TAP medium. The median value is represented by a solid horizontal line, with the boxes extending from the lower (25th percentile) to the upper (75th percentile) quartiles. The whiskers extend an interquartile range (IQR) from the mean, which is indicated by a hollow square marker. The data were compiled from a single microcolony, comprising of 600 cells in total. (C) Real fluorescent images corresponding to each division onset episode are shown, highlighting parent cells prior to division and daughter cells following division completion. (D) Representative fluorescence images from three individual microcolonies captured at the onset of the fifth cell division cycle. Each cell is overlaid with a colour map corresponding to its cross-sectional area, with the colour scale shown at right. These images illustrate the spatial heterogeneity in cell size distribution within colonies at equivalent division stages.

The variation in mitotic rate across cell cycles and radial positions was also reflected in the size of parent cells prior to division. Each successive cell cycle was associated with distinct time points following the onset of imaging: 24 h (cycle 1), 49 h (cycle 2), 73 h (cycle 3), 98 (cycle 4), and 121 h (cycle 5). The average size of parent cells decreased progressively with each cell cycle, regardless of radial location within the microcolony (Fig. 2A). When stratified by radial distance from the colony centre (0–175 μm, colour-coded), it was observed that parent cells located near the centre generally exhibited smaller cross-sectional areas than those at the periphery, particularly during early cycles when the colony radius remained below ~100 μm (Fig. 2A and C).

On the other hand, the size of daughter cells remained relatively constant across division cycles, showing no clear monotonic trend with respect to radial position within the colony. This consistency in daughter cell size was further supported by the lack of correlation between the size of daughter cells and their corresponding parent cells, as indicated by a Spearman’s rank correlation coefficient (R_s_ = 0.0755) (Fig. 2B and Figure S9), regardless of cell cycle sequence and radial position within the microcolony. Representative bright-field images (Fig. 2C) depict colony growth over successive division cycles, showing increasing colony size and cell number over time.

**Figure 3:**
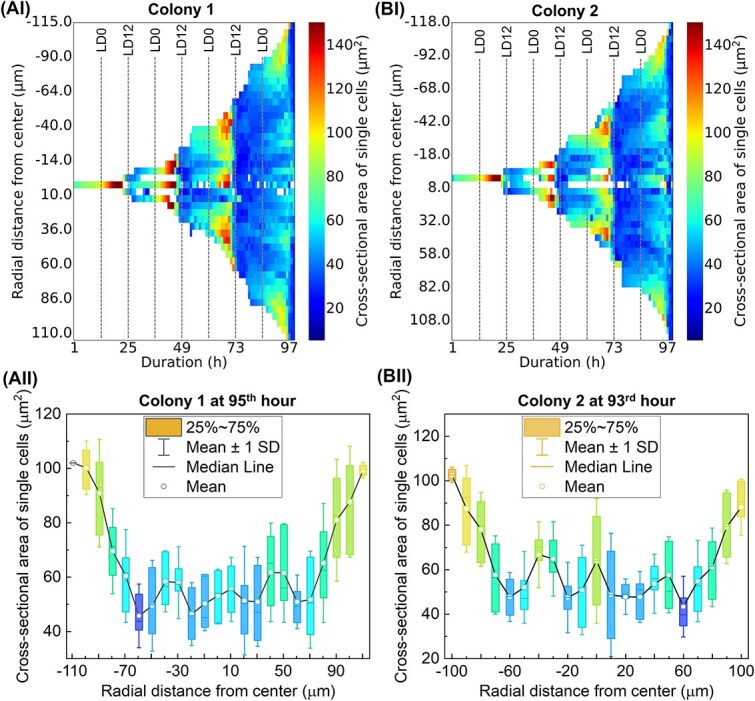
Spatiotemporal cell size dynamics in two independent microcolonies. (A, B) (i) radial kymographs and (ii) corresponding radial box plots of single-cell size measurements in two representative microcolonies (A) and (B). (i) Mean cell size over time and radial distance is visualised as a heatmap. Cells closer to the colony centre are positioned near the centre of the *y*-axis. Colour intensity represents the mean cross-sectional area of cells in each spatiotemporal bin. Vertical dashed lines indicate the timepoints of light–dark (LD12) and dark–light (LD0) transitions. Both colonies exhibit concentric, radially propagating waves of cell size, as seen by the periodic banding patterns moving outward from the centre. (ii) Distribution of single-cell areas across radial positions at a fixed time point, i.e. onset of the 4th division episode. Each box represents the interquartile range (25%–75%), with whiskers showing ±1 standard deviation from mean (SD), median line in orange, and white circles denoting the mean. Box fill colour corresponds to the mean cell size in each radial bin (mapped to the jet colormap). Periodic spatial fluctuations in cell size are observed in both colonies, supporting the presence of ring-like organisational structures.

While cells located in the microcolony core exhibited a phase lag in growth and division relative to peripheral cells, the relationship between cell size and radial position was not linear once the colony expanded beyond ~100 μm in diameter. Instead, an oscillatory pattern emerged, characterised by waves of cell size variation over time and space, radiating outward from the colony centre (Fig. 2D and Fig. 3). For instance, in the fifth cell cycle, cells located in the innermost ring (0–25 μm) and the mid-peripheral ring (100–125 μm) were smaller, while those in the third (50–75 μm) and outermost (150–175 μm) rings were comparatively larger (Fig. 2A). Additionally, deviations from a monotonic or linear trend in cell size distribution were observed in other microcolonies (Fig. 2D), highlighting spatial heterogeneity in division behaviour across colonies of similar developmental stages. To assess whether concentric patterns of cell size are reproducible across colonies, we analysed two independent microcolonies (Fig. 3A and B). Radial kymographs revealed dynamic, wave-like cell size patterns that originated near the centre and propagated outward over time. These spatiotemporal patterns manifested as alternating bands of larger and smaller cells aligned in concentric zones. Quantitative box plots of cell size at a fixed time point (onset of the 4th division) further confirmed these observations (Fig. 3ii). In both colonies, cell size varied nonmonotonically with radial distance, displaying periodic peaks and troughs. This spatial heterogeneity was consistent with concentric ring structures and suggests a robust, coordinated mechanism of radial growth regulation.

**Figure 4:**
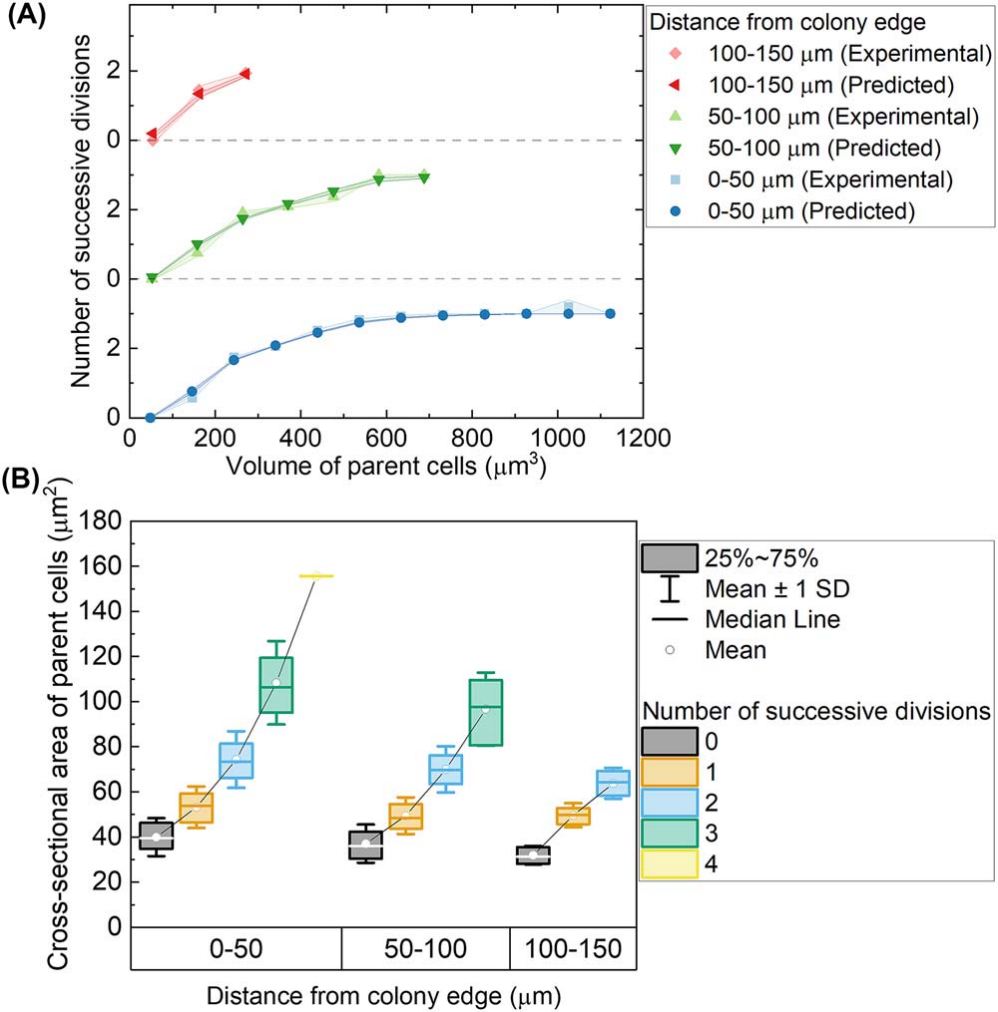
Mitotic sizer determines the number of daughter cells. (A) Comparison of observed and predicted number of successive divisions as a function of parent cell volume for different distance groups from the colony edge, evaluated using microscopic images at respective sequence of cell cycle, as compared to the values predicted with mitotic sizer modified threshold model [6]. Error bars indicate standard errors of the mean (SEM). Mother cell volume was binned, and the mean number of divisions ± SEM is shown for each bin. (B) Box-and-whisker plots showing the distribution of parent cell cross-sectional areas as a function of the number of daughter cells produced during division, across successive division cycles. Each data point represents an individual parent cell. Boxes represent the interquartile range (25th–75th percentile), with the median shown as a horizontal line and the mean indicated by a white circle. Whiskers represent ±1 standard deviation (SD). Data are grouped by the number of daughter cells (2, 4, 8, or 16), as indicated by colour, and plotted separately for each division cycle. (*n* = 21 microcolonies).

To ascertain whether the mitotic sizer rule was consistent across all sequential cycle numbers within the microcolony, the number of daughter cells from parent cells of different sizes, measured at various radial distances from the edge of the cell microcolony, was first evaluated using microscopic images (Fig. 4A). These experimental measurements were then compared to the predicted values derived from the modified threshold model of mitotic sizer described in [6]. To estimate cell volumes from two-dimensional time-lapse images, cells were approximated as ellipsoidal objects. The projected cross-sectional area was measured from fluorescence segmentation, and the minor axis length was assumed to represent the z-dimension of the ellipsoid. This approach follows the extrapolation method previously reported by Liu *et al.* [6]. The analysis revealed a good fit with the mitotic sizer model across all radial positions within the cell microcolony, indicating that the mechanism of the mitotic sizer remains unaffected and consistent regardless of the cells’ radial distance from the microcolony centre. The parameters of the modified threshold model are listed in Table S1. This hypothesis is further supported by the box plots illustrating the relationship between the number of daughter cells produced and the cross-sectional area of parent cells (Fig. 4B). The data showed that, across all radial distances from the centre of the cell microcolony, parent cells with sizes ranging from 40 to 70 μm^2^ typically underwent a single division, resulting in two daughter cells. Parent cells sized between 70 and 100 μm^2^ underwent two successive divisions. Cells with even larger sizes experienced three successive divisions. For maximum accuracy, the division numbers associated with parent cell sizes were determined manually through careful frame-by-frame inspection of the time-lapse data.

### Dependence of cell cycle upon hydrogel matrix

**Figure 5:**
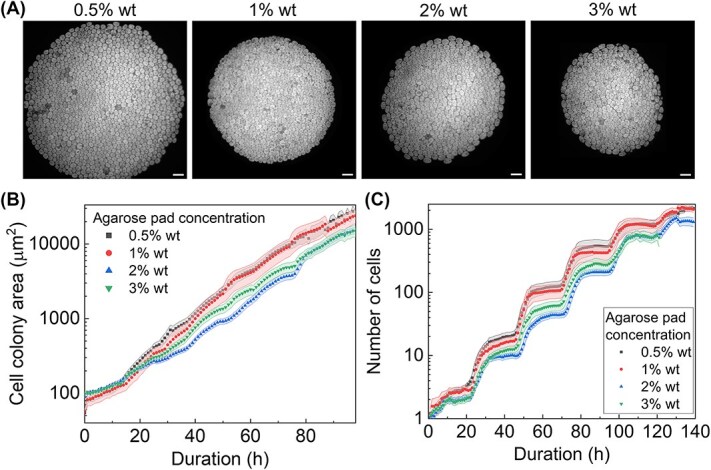
Agarose concentration affects microcolony development. (A) Representative microcolonies of *C. reinhardtii* captured beneath agarose gel pads of concentration 0.5% wt, 1% wt, 2% wt, and 3% wt imaged at the 120th hour. The scale bars correspond to 20 μm. (B) Expansion of microcolony area over time under varying agarose concentrations. Each point represents the average area across independent colonies per condition, with error band indicating standard error of mean (0.5% wt: *n* = 20; 1% wt: *n* = 10; 2% wt: *n* = 30; 3% wt: *n* = 30 microcolonies). (C) Plot illustrating cell number dynamics over time under varying gel concentrations, with the error band indicating standard error computed from 10 to 15 microcolonies in each condition. The dataset was compiled from two biological replicates (independent imaging runs), including 20 microcolonies grown in 0.5% agarose, 10 in 1%, 30 in 2%, and 30 in 3% agarose.

Here, we characterised the cell cycle under varied concentrations of gel matrix as a proxy for studying the varying degree of mechanical stress imposed on confined cells by the surrounding matrix. Generally, the mechanical stiffness of a gel pad scales linearly with the concentration of agarose in weight percentage [42]. Microcolonies were established between agarose gel pads and between 10 and 15 microcolonies were analysed for each gel concentration. After 5 days, the size of microcolonies demonstrated a negative correlation with agarose gel concentration (Fig. 5A). Cell number increase was the fastest with the lowest gel concentration (0.5% wt), while slower growth exhibited as the gel concentration increased up to 3% (Fig. 5B). For example, at the 110th hour, microcolonies under 0.5% wt and 1% wt agarose averaged 1200 cells, whereas those under 2% wt and 3% wt agarose averaged 800 cells. The distinction was reflected in the early stage of microcolony development at the 50th hour. Nonetheless, step-like features aligned with the diurnal cycle were seen at each agarose concentration (Fig. 5C). To examine variability across colonies, the coefficient of variation (CV) in colony size was also calculated at multiple time points (Fig. S10). These results indicate that there is no consistent trend in CV with increasing gel concentration. Instead, the observed variability appears to arise largely from heterogeneity in the timing of the first cell division event. In some colonies, division occurred early (within the first 10 h), while in others, division was delayed until after 24 h. This difference in initial proliferation timing resulted in substantial divergence in colony size at later time points, regardless of agarose concentration.

**Figure 6:**
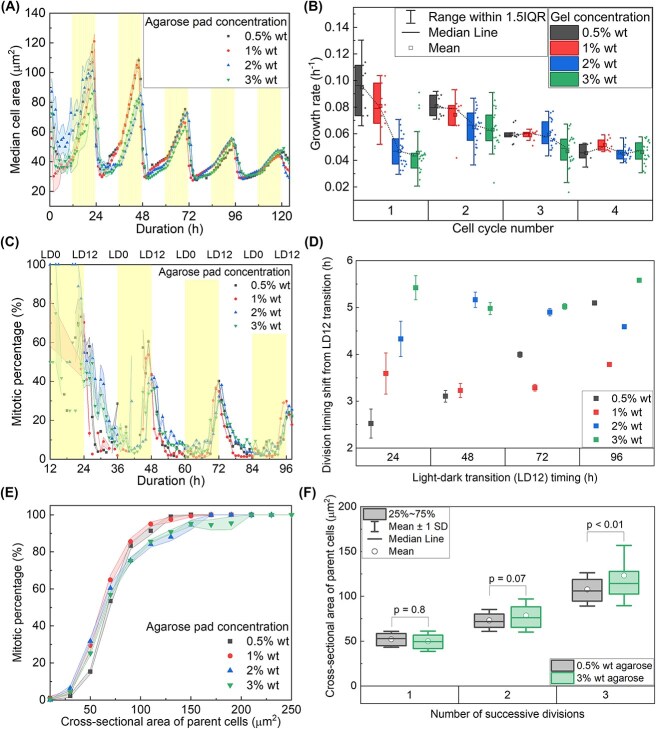
Mechanics of the environment influence growth rates but preserve sizer control robustness. (A) The temporal variation of the median cross-sectional cell area under agarose gel pads of varying concentrations is depicted, with error bands representing the standard error. This error was computed from analyses of 10 and 15 microcolonies for each gel concentration, respectively, with each microcolony containing over 1000 cells. The light phase is indicated by yellow shading, highlighting periods of illumination. (B) Dependence of cell growth rate on the sequence of cell cycles undertaken and the concentration of the agarose gel pad. The median value is represented by a solid horizontal line, with the boxes extending from the lower (25th percentile) to the upper (75th percentile) quartiles. The whiskers extend 1.5 times the interquartile range (IQR) from the upper and lower quartiles, and the mean is indicated by a hollow square marker. Each data point represents a single microcolony imaged. (C) The evolution of mitotic percentage within the microcolony population under a 12-h cycle of light (yellow shading)-dark (white) period. (D) Shift in cell division timing relative to the light–dark transition (LD12) under varying agarose concentrations. The *x*-axis indicates the time of the LD12 transition during the imaging process. The *y*-axis represents the deviation (in hours) between actual division onset and the LD12 transition, effectively capturing the shift in timing for each division event. Data points represent the mean timing shift for cells that produced 2, 4, 8, or 16 daughter cells, grouped by agarose concentration (0.5%, 1%, 2%, and 3% w/v). Error bars indicate standard error of mean (0.5% wt: *n* = 20; 1% wt: *n* = 10; 2% wt: *n* = 30; 3% wt: *n* = 30 microcolonies). (E) The dependence of mitotic percentage on the size of cells, beneath agarose gel pad of varied concentrations. The error band indicates standard error of mean (0.5% wt: *n* = 20; 1% wt: *n* = 10; 2% wt: *n* = 30; 3% wt: *n* = 30 microcolonies). Mitotic percentage was calculated using consecutive frames selected around the onset of division timepoints. (F) Box-and-whisker plots showing the distribution of parent cell cross-sectional areas as a function of the number of successive divisions, under 0.5% wt and 3% wt agarose, respectively. Boxes represent the interquartile range (25th–75th percentile), with the median shown as a horizontal line and the mean indicated by a white circle. Whiskers represent ±1 standard deviation (SD). Statistical comparisons between gel conditions at each division number were performed using one-way ANOVA followed by Tukey’s post hoc test, with p-values indicated above the brackets. The data correspond to the measurements summarized in Supplementary Table S3.

We next showed that the cell cycle progressed in accordance with diurnal conditions, maintaining regular intervals of around 24 h (Fig. 6A). The size of daughter cells remained consistent across all sequential cycles and was independent of gel concentration, as indicated by the convergence of minimum median cell area values at each division point. These results suggest that agarose concentration does not affect the size of daughter cells following cell division.

To show how these data provide insights into systems level dynamics of how mechanical pressure and light conditions affect cell size over time, an exponential model was fitted to the cell size variation data, determining the growth rate for each sequential cycle number under varying gel concentrations [7]:


(1) \begin{equation*} A(t)=A(0)\exp \left(\mu t\right) \end{equation*}

where $A(0)$ denotes the initial cell size and $\mu$ represents the rate of cell growth. In the initial three cell cycles, the growth rate decreased with increasing gel concentration (Fig. 6B). However, beyond the third cycle, cell growth rates across different gel concentrations did not display a notable trend. This convergence of growth rates across all gel conditions beyond third cycle could be attributed to the higher rate of cell growth and subsequent division numbers in the first three cycles, which resulted in a significantly higher cell density under lower gel concentrations. This is evident as the cell growth rates gradually decreased over successive cell cycles, regardless of agarose gel concentration (Fig. 6B).

There was a notable spread of mitotic events around the timepoint of the light–dark transition, with a small proportion of cells entering division before the peak timing (Fig. 6C). The earlier division was observed amongst cells of larger sizes [43]. Conversely, under higher gel concentrations, the division events extended well beyond the light–dark transition (LD12) into the dark phase, occurring after cells in lower gel concentrations had completed mitosis. This phenomenon was further evidenced by the mitotic percentage within the microcolonies at each division time point (Fig. 6C and Fig. S11), where a higher gel concentration corresponded to a lower fraction of cells entering the mitotic phase by the end of the light phase. Additionally, the average timing of cell division shifted progressively away from the LD12 transition as the gel concentration increased (Fig. 6D), indicating that a stiffer mechanical environment delays the coordination of mitotic entry with the light–dark cycle. The plot of mitotic percentage versus parent cell size also indicates the consistency of the commitment size across all gel concentrations (Fig. 6E). The mitotic percentage increased linearly with parent cell size up to 75 μm^2^, beyond which the slope of increase became gentler. Given the same parent cell size, the mitotic rate decreased slightly with increasing gel concentration. The number of successive divisions produced was positively associated with the cross-sectional area of the parent cell, regardless of the surrounding gel concentration (Fig. 6F). While parent cell sizes were comparable at division number 1 and 2 across both 0.5% and 3% agarose gels (*P* > 0.05), cells in 3% agarose exhibited significantly larger parent sizes than those in 0.5% agarose by the third division (*P* < 0.01) (Fig. 6F). For maximum accuracy, the division numbers associated with parent cell sizes were determined manually through careful frame-by-frame inspection of the time-lapse data.

## Discussion

*C. reinhardtii* cells typically grow during the light phase and divide during the dark phase under diurnal conditions, resulting in apparent synchrony. This synchrony is imposed by the light–dark cycle, while the actual timing of division is governed by intrinsic size and timer controls that operate independently of external cues [2]. While classical synchronisation protocols for *C. reinhardtii* typically employ autotrophic conditions such as high salt medium [44], recent studies have demonstrated that synchronisation can also be achieved under mixotrophic conditions using TAP medium supplemented with acetate, provided that appropriate light–dark cycles are maintained [45], as demonstrated in this work (Figs 1A and 6A). As microcolonies matured, however, not all cells continued to grow and divide in strict accordance with this cycle. Over time, cell cycle synchrony diminished, exhibiting a distinctive spatial pattern across the microcolony (Fig. 1). A progressive phase delay relative to the light–dark cycle was observed during colony expansion, potentially influenced by position-dependent factors such as mechanical constraints or cell–cell contact [22], though further work is needed to determine causality.

The linear increase in parent cell size with radial distance from the colony centre began to break down once the colony exceeded a certain size threshold (Fig. 3). At this stage, a concentric, wave-like pattern in cell size emerged, which we interpret as a manifestation of localized mechanical stress arising from the collective macroscopic growth of all cells within the confined microcolony. This interpretation is supported by discrete particle simulations and analytical models that predict similar spatial structuring under mechanical pressure in growing cell populations [46]. With a lower level of mechanical stress and greater access to nutrients or gaseous requirements [47–49], cells in the periphery tend to be more active with higher growth and division rates [50], leading to a higher number density of cells per unit area. Conversely, centrally located cells experiencing greater mechanical stress displayed reduced growth rates [51]. While contact inhibition amongst densely packed cells remains a possible contributing factor, no direct measurements of mechanical signalling were performed in this study. The increasing impact of cell crowding and diffusion limitations across microcolonies was also evident in the reduction of cell growth rates over time under 0.5% wt and 1% wt agarose gel pads (Fig. 6B).

When cells are confined in tight clusters, the cell cycle can be influenced by a combination of factors, including restricted access to essential substrates. The availability of nutrients such as nitrogen, phosphorus, sulfur, and carbon dioxide for photosynthesis may be compromised due to diffusion limitations, particularly for cells located farther from the colony surface or nutrient source [52, 53]. In such cases, localized deficiencies can arise, potentially triggering cell cycle arrest and entry into a quiescent state [54]. Additionally, the diffusion limitation of metabolic byproducts can result in the accumulation of reactive oxygen species, causing oxidative damage [55]. Furthermore, direct contact amongst neighbouring cells introduces physical constraints, such as space limitations and contact inhibition [56], which become more prominent in densely packed cell clusters [57]. With evidence of quorum sensing in *C. reinhardtii* [58, 59], there is likely a considerable accumulation of signalling molecules at high cell densities within closely packed microcolonies. Though localized heterogeneity in solute and gaseous concentrations within microcolonies cannot be entirely ruled out, this study argues against nutrient limitation as a primary driver of the observed spatial heterogeneity in cell size. Given the small size of the microcolonies analysed in this study—typically below 200 μm in diameter—and the early appearance of spatial heterogeneity in cell size, it is less likely that diffusion limitation was the dominant factor shaping growth dynamics at these early stages. This interpretation is consistent with observations from bacterial microcolonies, where aggregates of similar dimensions (≤200 μm in radius) have been shown to exhibit minimal nutrient depletion and maintain growth rates comparable to planktonic cultures under nutrient-rich conditions [52]. While differences in physiology between bacteria and microalgae exist, the physical constraints on diffusion in dense cellular aggregates are broadly comparable, particularly with respect to small solutes such as oxygen (O₂), carbon dioxide (CO₂), nitrogenous compounds (e.g. nitrate, ammonium), or phosphate [53]. Thus, while localised gradients of gases such as O₂ or CO₂ may still arise, the scale and timing of cell size divergence suggest that mechanical factors or cell–cell interactions are more likely contributors in the early phases of colony development. Importantly, this interpretation does not exclude the potential influence of diffusion-limited substrate availability; rather, it highlights that diffusion constraints alone are unlikely to account for the observed spatial heterogeneity, particularly at early time points and small colony sizes.

Beyond a radius of 200 μm, diffusion limitations become progressively more significant, leading to the formation of internal gradients and heterogeneous growth behaviour. Importantly, Jeanson *et al.* [52] focused on three-dimensional bacterial aggregates, where diffusion distances are inherently longer and more restrictive. In contrast, the present study investigates planar microcolonies, where cells are predominantly arranged in two dimensions. This reduced dimensionality facilitates more efficient diffusion of solutes and gases across the colony, further minimizing the likelihood of significant internal nutrient gradients at the small colony scales examined. To further evaluate the potential role of diffusion limitation within microcolonies, oxygen concentration profiles were measured across *C. reinhardtii* colonies of different sizes. In colonies with diameters approaching 600 μm, a visible oxygen gradient was observed, with higher oxygen concentrations detected toward the core of the colony and closer to the light source (Fig. S12). In contrast, in smaller colonies with diameters around 200 μm, oxygen concentrations remained relatively uniform across both lateral and vertical dimensions, with no significant accumulation at the centre under illumination or significant deficiency under darkness (Fig. S13). These findings demonstrate that while oxygen diffusion limitations can develop in larger, densely packed aggregates, microcolonies of the size range analysed in the present study are unlikely to experience substantial oxygen or nutrient depletion. Thus, the spatial heterogeneity in growth and division observed is more likely attributed to mechanical and spatial factors, rather than to localized resource limitation.

Spatiotemporal analysis of cell size variation across individual colonies revealed the impact of physical confinement and mechanical pressure imposed by cell crowding, upon the mechanisms of commitment and mitotic sizers within colonies. The commitment threshold remains consistent across spatial locations within the colony, with no variation in the slope gradient (Fig. 1D and S8). On the other hand, higher gel matrix concentration and thus mechanical stiffness led to lower rate of cell growth and divisions (Fig. 6B and C). Since cells are only able to enter the mitotic phase once they reached a size beyond the commitment point, when this size threshold was not achieved, the timing of division was delayed until the next diurnal cycle. Rather than dividing at a smaller cell size, cell division was postponed until sufficient growth was achieved. Delayed division provides additional time for biomass accumulation, leading to progressively larger parent cells over successive cycles (Fig. 6F). The number of daughter cells produced was solely dependent on the parent cell size, regardless of the gel concentration. This phenomenon, where increased gel matrix stiffness led to a reduction in overall cell size and productivity, has been reported in other studies of photosynthetic microorganisms [60, 61]. Furthermore, the reduction in diffusivity and permeability with higher agar concentrations inhibited bacterial microcolony expansion with slower fluid transport with nutrients [62, 63].

After the attainment of commitment threshold, once cell division was initiated, regulation by the mitotic sizer remained unaffected by the gel concentrations, as reflected in the consistent relationship between parent cell size and number of daughter cells across all gel concentrations (Figs 4B and 6F). Similarly, within an individual microcolony, the size of daughter cells remained relatively constant, showing only slight variations depending on the parent cell size, regardless of spatial position within the microcolony (Fig. 2). These findings reinforce the consistency of the mitotic sizer mechanism across varying spatial contexts within the microcolony. This suggests that the regulation of cell size at mitosis is robust and uniformly maintained throughout the microcolony.

It is also important to highlight that the cells immobilised within the gel pad, although closely clustered together, do not exhibit the typical stress response associated with palmelloid formation in *C. reinhardtii* [64, 65]. In the palmelloid state, cells do not fully divide and are encapsulated within a persistent outer cell wall envelope derived from the parent cell [66]. Additionally, cells in the palmelloid state possess truncated, dysfunctional, or entirely absent flagella [67]. In contrast, cells immobilised in the gel pad displayed fully developed flagella, which were clearly visible around the edges of microcolonies (Fig. S14). These cells retained motility and regained their swimming ability once the gel was removed from the substrate.

Studying confined microcolonies of *C. reinhardtii* provided new insights into the effects of cell crowding on the cell cycle and offers a model for investigating initial biofilm formation on a reduced scale. The observed spatial dependence of phase lag in cell cycle progression amongst confined cells mirrors, on a reduced scale, the spatial partitioning of biofilms into growing and resting regimes [24]. In the proposed mechanism, cells positioned at the upper surface of the biofilm, furthest from the solid substrate, are in the growing regime. The microcolonies formed a largely planar structure with increased vertical layering near the centre, resulting in central cells being located beneath multiple overlying layers and experiencing greater light shading compared to peripheral cells. As illumination was applied from above, peripheral cells—closer to the surface—were more exposed to light, whereas centrally located cells, especially those near the base of the dome, likely experienced reduced light availability. In dense algal clusters, even a few layers of cells can significantly attenuate incident light, potentially limiting photosynthetic activity and slowing division rates in the inner regions of the colony (Fig. S12b) [68]. These findings may also have relevance for understanding how *C. reinhardtii* coordinates growth and division in spatially confined microenvironments, such as soil pores or on particle surfaces [69, 70]. In such habitats, cells may experience physical constraints, heterogeneous access to light and nutrients, and dense local populations—all of which may influence cell cycle dynamics in ways similar to those observed in our immobilised colony system. While the results in this work underscore the importance of mechanical and spatial cues in modulating cell cycle dynamics, extrapolation to more complex environments, such as biofilms or natural consortia, requires caution. Future work incorporating additional environmental variables (e.g. nutrient gradients, light availability, extracellular matrix components) will be critical for assessing the generalizability of these results. Nevertheless, the framework established here offers a versatile and scalable platform for such investigations, particularly in controlled comparative analyses of wild-type and mutant strains. While the present study focused on wild-type strains, the platform is well-positioned to analyse quantitative phenotypes in nonlethal mutants, particularly those affecting the mitotic sizer system (e.g. *mat3*, *dp*, *cdka*) [6]. These mutants, which alter cell size control without compromising viability, represent a valuable opportunity for future studies to leverage our colony-level growth analysis to uncover novel insights into cell cycle regulation.

A limitation of this study is the absence of direct viability assays, such as trypan blue exclusion or propidium iodide staining, to explicitly quantify cell death or complete growth arrest. Furthermore, subtle forms of metabolic stress or nonlethal arrest may not be fully captured by this current approach, and future studies incorporating direct viability assessments would be valuable in complementing the findings. In future applications, the platform could be extended through the use of multichannel fluorescence imaging to probe additional cellular processes beyond growth and division. For example, fluorescent reporters could be employed to monitor cell cycle regulators, stress responses, metabolic states, or signalling dynamics in real time [12, 71, 72]. Similarly, photosynthetic capacity could be assessed through variable chlorophyll fluorometry techniques—such as pulse-amplitude modulatio or fast repetition rate fluorometry—to provide spatially resolved measurements of photophysiological performance within microcolonies [73, 74]. Such additions would enable simultaneous tracking of molecular, physiological, and phenotypic features at the single-cell level within a spatially structured context, further enhancing the platform’s utility for dissecting complex developmental and stress-response behaviours in immobilised microalgal systems [75].

Although this study focused on population-level and spatial trends in cell division, the potential value of lineage-level analysis is recognised. Due to the 1-h imaging interval employed, long-term monitoring of multiple colonies was achieved; however, this temporal resolution was not sufficient to accurately resolve the timing and sequence of individual division events across full lineages. As a result, the reconstruction of complete division histories was limited. Future studies will aim to incorporate higher temporal resolution imaging in selected fields of view, allowing more detailed tracking of individual cells and their progeny. When combined with refined segmentation and automated tracking workflows, this approach is expected to support high-confidence lineage reconstruction. Such developments would enable the investigation of lineage-dependent variability, heritability of division timing, and the influence of spatial positioning on cell cycle progression under confinement.

In conclusion, this work demonstrates how cell cycles vary within individual colonies, the effect of surrounding matrix on the cell cycle progressions, and how the commitment and mitotic sizer are affected as a consequence. As microalgal microcolonies mature, the typical light-dependent growth and synchronous division cycle becomes less consistent, with cell cycle synchrony diminishing and displaying a distinctive spatial pattern. Despite these variations, daughter cell size remains relatively consistent. A notable reduction in cell growth rate and delay in cell divisions was observed with increasing gel concentration, yet the regulation of the mitotic sizer and light-gated timer mechanisms remained unaffected. These findings offer a framework for understanding how physical and environmental constraints affect cellular behaviour, advancing models for biohybrid systems and microalgal biotechnologies.

## Supplementary Material

20250615_ISME_Communications_SI_ycaf104

## Data Availability

All custom code used for image preprocessing, segmentation, and statistical analysis is publicly available via the Zenodo repository: https://doi.org/10.5281/zenodo.14514702. Experimental datasets supporting the findings of this study are also deposited in the same repository.

## References

[1] Cross FR, Umen JG. The *Chlamydomonas* cell cycle. *Plant J* 2015;82:370–92. 10.1111/tpj.12795 25690512 PMC4409525

[2] Zachleder V, Ende HVD. Cell cycle events in the green alga *Chlamydomonas eugametos* and their control by environmental factors. *J Cell Sci* 1992;102:469–74. 10.1242/jcs.102.3.469

[3] Harper JDI, Wu L, Sakuanrungsirikul S et al. Isolation and partial characterization of conditional cell division cycle mutants in *Chlamydomonas*. *Protoplasma* 1995;186:149–62. 10.1007/BF01281325

[4] Vítová M, Bišová K, Hlavová M et al. *Chlamydomonas reinhardtii*: duration of its cell cycle and phases at growth rates affected by temperature. *Planta* 2011;234:599–608. 10.1007/s00425-011-1427-7 21573815

[5] Vítová M, Bišová K, Umysová D et al. *Chlamydomonas reinhardtii*: duration of its cell cycle and phases at growth rates affected by light intensity. *Planta* 2011;233:75–86. 10.1007/s00425-010-1282-y 20922544

[6] Liu D, Vargas-García CA, Singh A et al. A cell-based model for size control in the multiple fission alga *Chlamydomonas reinhardtii*. *Curr Biol* 2023;33:5215–24.e5. 10.1016/j.cub.2023.10.023 37949064 PMC10750806

[7] Matsumura K, Yagi T, Hattori A et al. Using single cell cultivation system for on-chip monitoring of the interdivision timer in *Chlamydomonas reinhardtii* cell cycle. *J Nanobiotechnol* 2010;8:23–3. 10.1186/1477-3155-8-23 PMC2955706 20868509

[8] Donnan L, John PCL. Cell cycle control by timer and sizer in *Chlamydomonas*. *Nature* 1983;304:630–3. 10.1038/304630a0 6877383

[9] Matsumura K, Yagi T, Yasuda K. Role of timer and sizer in regulation of *Chlamydomonas* cell cycle. *Biochem Biophys Res Commun* 2003;306:1042–9. 10.1016/S0006-291X(03)01089-1 12821148

[10] Hu Q, Sommerfeld M, Jarvis E et al. Microalgal triacylglycerols as feedstocks for biofuel production: perspectives and advances. *Plant J* 2008;54:621–39. 10.1111/j.1365-313X.2008.03492.x 18476868

[11] Rosenthal K, Oehling V, Dusny C et al. Beyond the bulk: disclosing the life of single microbial cells. *FEMS Microbiol Rev* 2017;41:751–80. 10.1093/femsre/fux044 29029257 PMC5812503

[12] Hardo G, Bakshi S. Challenges of analysing stochastic gene expression in bacteria using single-cell time-lapse experiments. *Essays Biochem* 2021;65:67–79. 10.1042/EBC20200015 33835126 PMC8056041

[13] Shih SCC, Mufti NS, Chamberlain MD et al. A droplet-based screen for wavelength-dependent lipid production in algae. *Energy Environ Sci* 2014;7:2366–75. 10.1039/c4ee01123f

[14] Westerwalbesloh C, Brehl C, Weber S et al. A microfluidic photobioreactor for simultaneous observation and cultivation of single microalgal cells or cell aggregates. *PLoS One* 2019;14:e0216093–3. 10.1371/journal.pone.0216093 31034529 PMC6488086

[15] Luke CS, Selimkhanov J, Baumgart L et al. A microfluidic platform for long-term monitoring of algae in a dynamic environment. *ACS Synth Biol* 2016;5:8–14. 10.1021/acssynbio.5b00094 26332284 PMC5249263

[16] Kim HS, Weiss TL, Thapa HR et al. A microfluidic photobioreactor array demonstrating high-throughput screening for microalgal oil production. *Lab Chip* 2014;14:1415–25. 10.1039/c3lc51396c 24496295

[17] Bae S, Kim CW, Choi JS et al. An integrated microfluidic device for the high-throughput screening of microalgal cell culture conditions that induce high growth rate and lipid content. *Anal Bioanal Chem* 2013;405:9365–74. 10.1007/s00216-013-7389-9 24170268

[18] Behrendt L, Salek MM, Trampe EL et al. Phenochip: a single-cell phenomic platform for high-throughput photophysiological analyses of microalgae. *Science*. *Advances* 2020;6:eabb2754. 10.1126/sciadv.abb2754 PMC7467707 32917592

[19] Park JW, Na SC, Nguyen TQ et al. Live cell imaging compatible immobilization of *chlamydomonas reinhardtii* in microfluidic platform for biodiesel research. *Biotechnol Bioeng* 2015;112:494–501. 10.1002/bit.25453 25220860

[20] Lee D, Bae C, Han J et al. In situ analysis of heterogeneity in the lipid content of single green microalgae in alginate hydrogel microcapsules. *Anal Chem* 2013;85:8749–56. 10.1021/ac401836j 24007509

[21] Best R, Abalde-Cela S, Abell C et al. Applications of microdroplet technology for algal biotechnology. *Current Biotechnology* 2016;5:109–17. 10.2174/2211550105666160202002554

[22] Min S, Yoon GH, Joo JH et al. Mechanosensitive physiology of *Chlamydomonas reinhardtii* under direct membrane distortion. *Sci Rep* 2014;4:4675. 10.1038/srep04675 24728350 PMC3985077

[23] Moreno , Osorio JH, Pollio A, Frunzo L et al. A review of microalgal biofilm technologies: definition, applications, settings and analysis. *Frontiers*. *Chem Eng* 2021;3:737710. 10.3389/fceng.2021.737710

[24] Li L, Wang Y, Gao L et al. Experiments and cellular automata simulation reveal light/carbon transportation and growth mechanism of *Chlorella vulgaris* biofilm in attached cultivation. *Chem Eng J* 2023;457:141177. 10.1016/j.cej.2022.141177

[25] Caldwell G, In-na P, Hart R et al. Immobilising microalgae and cyanobacteria as biocomposites: new opportunities to intensify algae biotechnology and bioprocessing. *Energies* 2021;14:2566. 10.3390/en14092566

[26] Malik S, Hagopian J, Mohite S et al. Robotic extrusion of algae-laden hydrogels for large-scale applications. *Global Chall* 2020;4:1900064. 10.1002/gch2.201900064 PMC6957016 31956429

[27] Kennard AS, Osella M, Javer A et al. Individuality and universality in the growth-division laws of single *E. coli cells*. *Phys Rev E* 2016;93:012408. 10.1103/PhysRevE.93.012408 26871102

[28] Grant MAA, Wacław B, Allen RJ et al. The role of mechanical forces in the planar-to-bulk transition in growing *escherichia coli* microcolonies. *J R Soc Interface* 2014;11:20140400. 10.1098/rsif.2014.0400 24920113 PMC4208374

[29] Gorman DS, Levine RP. Cytochrome f and plastocyanin: their sequence in the photosynthetic electron transport chain of *Chlamydomonas reinhardi*. *Proc Natl Acad Sci* 1965;54:1665–9. 10.1073/pnas.54.6.1665 4379719 PMC300531

[30] Kropat J, Hong-Hermesdorf A, Casero D et al. A revised mineral nutrient supplement increases biomass and growth rate in *Chlamydomonas reinhardtii*. *Plant J* 2011;66:770–80. 10.1111/j.1365-313X.2011.04537.x 21309872 PMC3101321

[31] Schindelin J, Arganda-Carreras I, Frise E et al. Fiji: an open-source platform for biological-image analysis. *Nat Methods* 2012;9:676–82. 10.1038/nmeth.2019 22743772 PMC3855844

[32] Schmidt U, Weigert M, Broaddus C and Myers G. Cell detection with star-convex polygons. In:Frangi AF, Schnabel JA, Davatzikos C, Alberola-López C and Fichtinger G. (eds.). Medical Image Computing and Computer Assisted Intervention – MICCAI. 2018. Springer International Publishing (Cham, Switzerland). 265–73.

[33] Stringer C, Wang T, Michaelos M et al. Cellpose: a generalist algorithm for cellular segmentation. *Nat Methods* 2021;18:100–6. 10.1038/s41592-020-01018-x 33318659

[34] Carpenter AE, Jones TR, Lamprecht MR et al. Cellprofiler: image analysis software for identifying and quantifying cell phenotypes. *Genome Biol* 2006;7:R100. 10.1186/gb-2006-7-10-r100 17076895 PMC1794559

[35] Rickelt LF, Lichtenberg M, Trampe ECL et al. Fiber-optic probes for small-scale measurements of scalar irradiance. *Photochem Photobiol* 2016;92:331–42. 10.1111/php.12560 26715143

[36] Spilling K, Titelman J, Greve TM et al. Microsensor measurements of the external and internal microenvironment of *Fucus vesiculosus* (phaeophyceae). *J Phycol* 2010;46:1350–5. 10.1111/j.1529-8817.2010.00894.x

[37] Kühl M, Lassen C, Revsbech NP. A simple light meter for measurements of PAR (400 to 700 nm) with fiber-optic microprobes: application for p vs e_0_ (PAR) measurements in a microbial mat. *Aquat Microb Ecol* 1997;13:197–207. 10.3354/ame013197

[38] Lichtenberg M, Nørregaard RD, Kühl M. Diffusion or advection? Mass transfer and complex boundary layer landscapes of the brown alga *Fucus vesiculosus*. *J R Soc Interface* 2017;14:20161015. 10.1098/rsif.2016.1015 28330986 PMC5378137

[39] Wangpraseurt D, Holm JB, Larkum AWD et al. In vivo microscale measurements of light and photosynthesis during coral bleaching: evidence for the optical feedback loop? *Front Microbiol* 2017;8:59. 10.3389/fmicb.2017.00059 28174567 PMC5258690

[40] Zones JM, Blaby IK, Merchant SS et al. High-resolution profiling of a synchronized diurnal transcriptome from *Chlamydomonas reinhardtii* reveals continuous cell and metabolic differentiation. *Plant Cell* 2015;27:2743–69. 10.1105/tpc.15.00498 26432862 PMC4682324

[41] You Z, Pearce DJG, Sengupta A et al. Mono- to multilayer transition in growing bacterial colonies. *Phys Rev Lett* 2019;123:178001. 10.1103/PhysRevLett.123.178001 31702266

[42] Mori Y, Kanazawa S, Watanabe M et al. Usefulness of agarose mold as a storage container for three-dimensional tissue-engineered cartilage. *Mater Sci Appl* 2013;4:73–8. 10.4236/msa.2013.48A010

[43] Craigie RA, Cavalier-smith T. Cell volume and the control of the *Chlamydomonas* cell cycle. *J Cell Sci* 1982;54:173–91. 10.1242/jcs.54.1.173

[44] Hlavová M, Vítová M, Bišová K. Synchronization of green algae by light and dark regimes for cell cycle and cell division studies. In: Caillaud M.-C. (ed.), Plant Cell Division: Methods and Protocols. New York: NY: springer New York, 3–16, 10.1007/978-1-4939-3142-2_1 26659950

[45] Angstenberger M, de Signori F, Vecchi V et al. Cell synchronization enhances nuclear transformation and genome editing via cas9 enabling homologous recombination in *Chlamydomonas reinhardtii*. *ACS Synth Biol* 2020;9:2840–50. 10.1021/acssynbio.0c00390 32916053 PMC8011982

[46] Weady S, Palmer B, Lamson A et al. Mechanics and morphology of proliferating cell collectives with self-inhibiting growth. *Phys Rev Lett* 2024;133:158402. 10.1103/PhysRevLett.133.158402 39454152

[47] Welker A, Hennes M, Bender N et al. Spatiotemporal dynamics of growth and death within spherical bacterial colonies. *Biophys J* 2021;120:3418–28. 10.1016/j.bpj.2021.06.022 34214531 PMC8391034

[48] Maier B . How physical interactions shape bacterial biofilms. *Annu Rev Biophys* 2021;50:401–17. 10.1146/annurev-biophys-062920-063646 33637007

[49] Mahajan V, Beck T, Gregorczyk P et al. Mapping tumor spheroid mechanics in dependence of 3D microenvironment stiffness and degradability by brillouin microscopy. *Cancers (Basel)* 2021;13:5549. 10.3390/cancers13215549 34771711 PMC8583550

[50] Dell’Arciprete D, Blow ML, Brown AT et al. A growing bacterial colony in two dimensions as an active nematic. *Nat Commun* 2018;9:4190. 10.1038/s41467-018-06370-3 30305618 PMC6180060

[51] Wittmann R, Nguyen GHP, Löwen H et al. Collective mechano-response dynamically tunes cell-size distributions in growing bacterial colonies. *Commun Phys* 2023;6:331. 10.1038/s42005-023-01449-w

[52] Jeanson S, Floury J, Gagnaire V et al. Bacterial colonies in solid media and foods: a review on their growth and interactions with the micro-environment. *Front Microbiol* 2015;6:1284. 10.3389/fmicb.2015.01284 PMC4664638 26648910

[53] Stewart PS . Diffusion in biofilms. *J Bacteriol* 2003;185:1485–91. 10.1128/jb.185.5.1485-1491.2003 12591863 PMC148055

[54] Takeuchi T, Benning C. Nitrogen-dependent coordination of cell cycle, quiescence and tag accumulation in *Chlamydomonas*. *Biotechnol Biofuels* 2019;12:292. 10.1186/s13068-019-1635-0 31890020 PMC6927116

[55] Wakao S, Niyogi KK. *Chlamydomonas* as a model for reactive oxygen species signaling and thiol redox regulation in the green lineage. *Plant Physiol* 2021;187:687–98. 10.1093/plphys/kiab355 35237823 PMC8491031

[56] Moore KA, Altus S, Tay JW et al. Mechanical regulation of photosynthesis in cyanobacteria. *Nat Microbiol* 2020;5:757–67. 10.1038/s41564-020-0684-2 32203409

[57] Ikryannikova LN, Kurbatov LK, Gorokhovets NV et al. Contact-dependent growth inhibition in bacteria: do not get too close! *Int J Mol Sci* 2020;21:7990. 10.3390/ijms21217990 33121148 PMC7662968

[58] Teplitski M, Chen H, Rajamani S et al. *Chlamydomonas reinhardtii* secretes compounds that mimic bacterial signals and interfere with quorum sensing regulation in bacteria. *Plant Physiol* 2004;134:137–46. 10.1104/pp.103.029918 14671013 PMC316294

[59] Folcik AM, Cutshaw K, Haire T et al. Quorum sensing behavior in the model unicellular eukaryote *Chlamydomonas reinhardtii*. *iScience* 2020;23:101714. 10.1016/j.isci.2020.101714 33196031 PMC7644740

[60] Kumar V, Nanda M, Verma M. Application of agar liquid-gel transition in cultivation and harvesting of microalgae for biodiesel production. *Bioresour Technol* 2017;243:163–8. 10.1016/j.biortech.2017.06.080 28654837

[61] Dickson DJ, Page CJ, Ely RL. Photobiological hydrogen production from Synechocystis sp. PCC 6803 encapsulated in silica sol–gel. *Int J Hydrog Energy* 2009;34:204–15. 10.1016/j.ijhydene.2008.10.021

[62] Little K, Austerman J, Zheng J et al. Cell shape and population migration are distinct steps of *Proteus mirabilis* swarming that are decoupled on high-percentage agar. *J Bacteriol* 2019;201. 10.1128/jb.00726-18 PMC6509654 30858303

[63] Yan J, Nadell CD, Stone HA et al. Extracellular-matrix-mediated osmotic pressure drives *vibrio cholerae* biofilm expansion and cheater exclusion. *Nat Commun* 2017;8:327. 10.1038/s41467-017-00401-1 28835649 PMC5569112

[64] Lurling M, Beekman W. Palmelloids formation in *Chlamydomonas reinhardtii*: defence against rotifer predators? *ANN LIMNOL-INT J LIM* 2006;42:65–72. 10.1051/limn/2006010

[65] de Carpentier F, Lemaire SD, Danon A. When unity is strength: the strategies used by *Chlamydomonas* to survive environmental stresses. *CELLS-BASEL* 2019;8:1307. 10.3390/cells8111307 PMC6912462 31652831

[66] Oh J-J, Ammu S, Vriend VD et al. Growth, distribution, and photosynthesis of *Chlamydomonas reinhardtii* in 3D hydrogels. *Adv Mater* 2024;36:2305505. 10.1002/adma.202305505 37851509

[67] Yoshitomi T, Kaminaga S, Sato N et al. Formation of spherical palmelloid colony with enhanced lipid accumulation by gel encapsulation of *Chlamydomonas debaryana* nies-2212. *Plant Cell Physiol* 2020;61:158–68. 10.1093/pcp/pcz188 31589321

[68] Chua ST, Smith A, Murthy S et al. Light management by algal aggregates in living photosynthetic hydrogels. *Proc Natl Acad Sci* 2024;121:e2316206121. 10.1073/pnas.2316206121 38805271 PMC11161743

[69] Nakada T, Shinkawa H, Ito T et al. Recharacterization of *Chlamydomonas reinhardtii* and its relatives with new isolates from Japan. *J Plant Res* 2010;123:67–78. 10.1007/s10265-009-0266-0 19882207

[70] Mansour I, Hähnlein M, Minkewitz L et al. Spatial structure affects the establishment and persistence of closed microbial ecosystems. *bioRxiv* 2024.06.28.601237. 10.1101/2024.06.28.601237

[71] Di Caprio F, Pagnanelli F, Wijffels RH et al. Quantification of *Tetradesmus obliquus* (chlorophyceae) cell size and lipid content heterogeneity at single-cell level. *J Phycol* 2018;54:187–97. 10.1111/jpy.12610 29194643

[72] Sandmann M, Schafberg M, Lippold M et al. Analysis of population structures of the microalga *Acutodesmus obliquus* during lipid production using multi-dimensional single-cell analysis. *Sci Rep* 2018;8:6242. 10.1038/s41598-018-24638-y 29674634 PMC5908859

[73] Széles E, Nagy K, Ábrahám Á et al. Microfluidic platforms designed for morphological and photosynthetic investigations of *Chlamydomonas reinhardtii* on a single-cell level. *Cells* 2022;11:285. 10.3390/cells11020285 35053401 PMC8774182

[74] Trampe EL, Kolbowski J, Schreiber U et al. Rapid assessment of different oxygenic phototrophs and single-cell photosynthesis with multicolour variable chlorophyll fluorescence imaging. *Mar Biol* 2011;158:1667–75. 10.1007/s00227-011-1663-1

[75] Sun J, Gao L, Wang L et al. Recent advances in single-cell analysis: encapsulation materials, analysis methods and integrative platform for microfluidic technology. *Talanta* 2021;234:122671. 10.1016/j.talanta.2021.122671 34364472

