# Supplementary material for: Uncoupling growth and division in Chlamydomonas reinhardtii colonies: consistent cell cycle regulation under confinement
Source: ISME Commun. 2025 Jun 23;5(1):ycaf104. doi: 10.1093/ismeco/ycaf104 (PMC12306439; doi:10.1093/ismeco/ycaf104)
Supplement: 20250615_ISME_Communications_SI_ycaf104 [file 20250615_isme_communications_si_ycaf104.pdf]

Supplementary Materials for

Uncoupling growth and division in *Chlamydomonas reinhardtii* colonies: Consistent cell cycle regulation under confinement

Authors

Sing Teng Chua[1], Jurij Kotar[2], Michael Kühl[3], Alison G. Smith[4], Silvia Vignolini[1, 5], Pietro Cicuta[2,*]

Affiliations

[1]Yusuf Hamied Department of Chemistry, University of Cambridge, Cambridge, United Kingdom

[2]Cavendish Laboratory, University of Cambridge, Cambridge, United Kingdom

[3]Marine Biology Section, Department of Biology, University of Copenhagen, Helsingør DK-3000, Denmark

[4]Department of Plant Sciences, University of Cambridge, Cambridge, United Kingdom

[5]Department of Sustainable and Bio-inspired Materials, Max Planck Institute of Colloids and Interfaces, Potsdam, Germany

*Corresponding author

Email: pc245@cam.ac.uk

This PDF file includes:

Figs. S1 to S14
Table S1 to S3

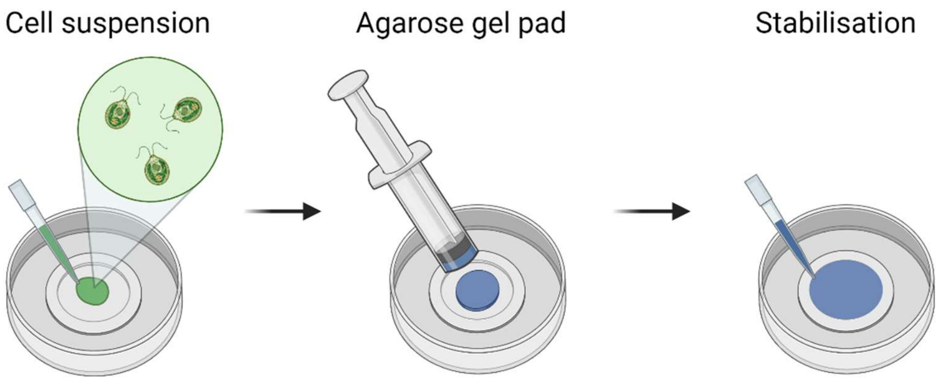

Fig. S1.
Immobilization of *C. reinhardtii* suspension for imaging purpose. An aliquot of cell suspension was sandwiched between the bottom coverslip and a thin flat agarose gel pad on top. A moat of molten gel surrounding the gel pad served to minimize lateral shift and potential gel lifting during constant stage movement.

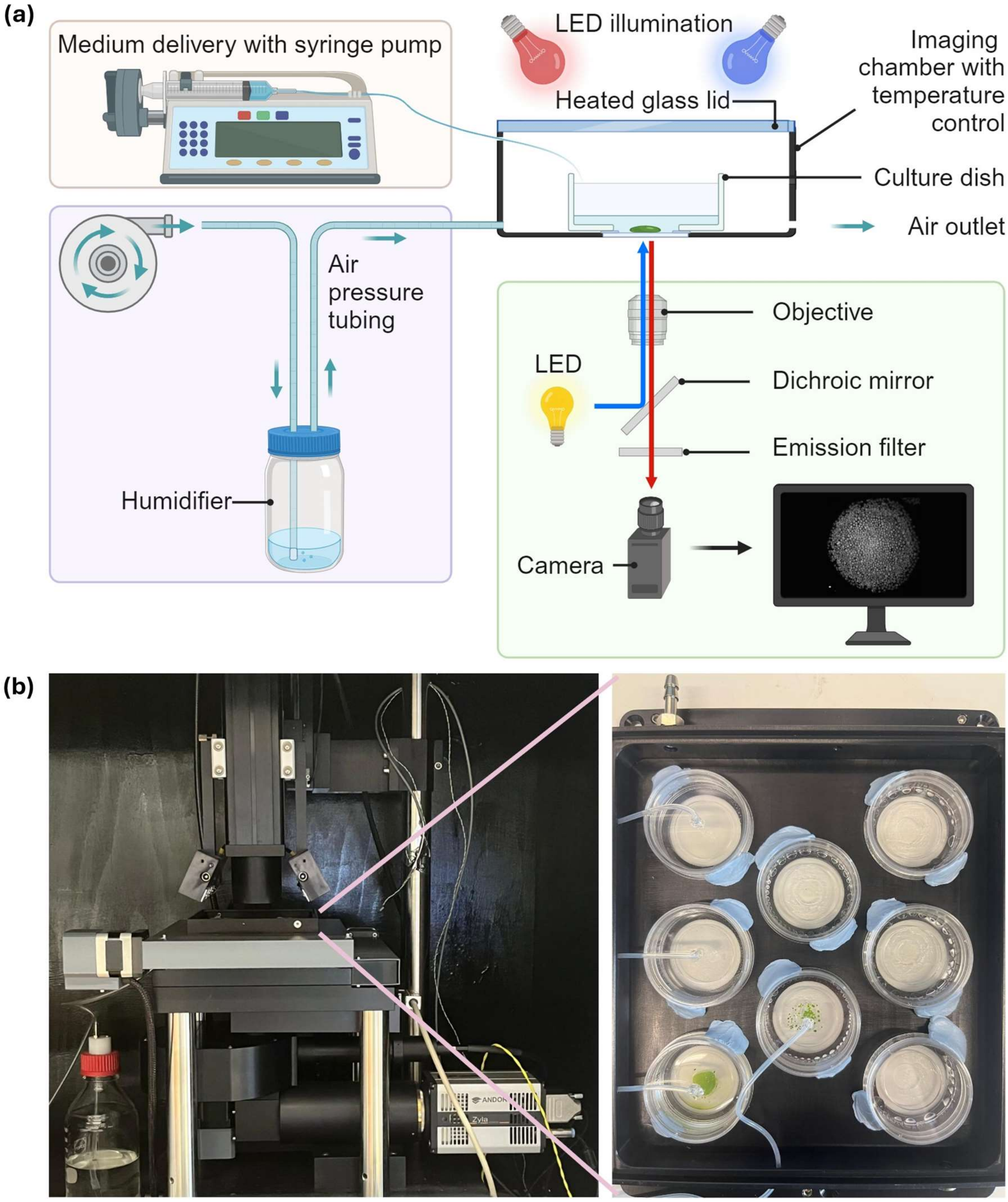

Fig. S2.

Experimental setup designed for time-lapse imaging and incubation of *C. reinhardtii* in commercial dishes (Ibidi µ-Dish 35 mm, 81151) with environmental control. (a) Schematics of the inverted epifluorescence microscopy setup and the incubation chamber, featuring humidity control and a nutrient delivery system. (b) A photograph of the black imaging chamber housing the inverted microscope, along with a close-up image showing the microscope stage equipped with a multi-well holder accommodating eight Ibidi dishes containing green microalgal microcolonies with nutrient supply tubings.

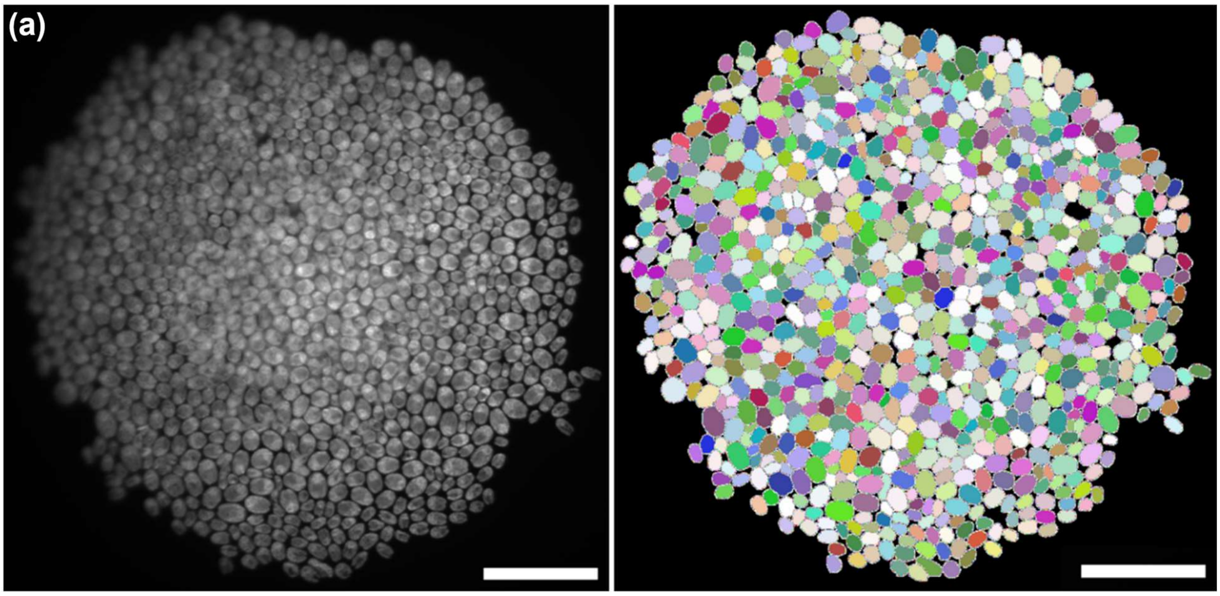

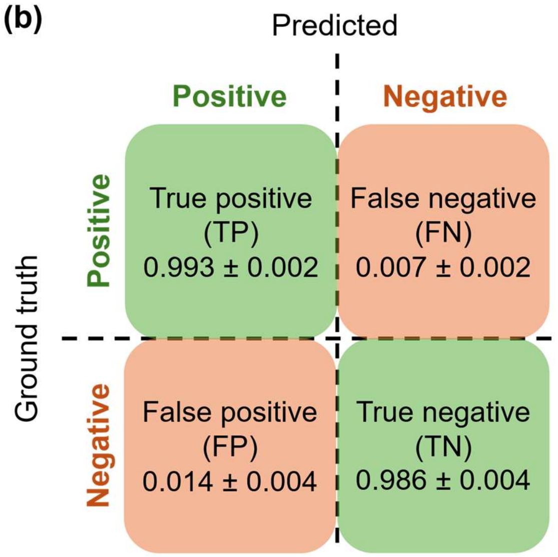

Performance metrics		
Precision	$\frac{TP}{TP + FP}$	0.989 ± 0.004
Sensitivity	$\frac{TP}{TP + FN}$	0.980 ± 0.006
Jaccard index (IoU)	$\frac{TP}{TP + FP + FN}$	0.980 ± 0.004
F1-score	$\frac{2TP}{2TP + FP + FN}$	0.984 ± 0.005

Fig. S3.

Evaluation of cell segmentation performance. (a) Representative image of a cell microcolony before and after cell segmentation using Stardist (scale bar = 50 um). (b) The performance metrics computed from true positive rate (TP), true negative rate (TN), false positive rate (FP) and false negative rate (FN) of pixel predictions. Positive pixels indicate the foreground area occupied by cells, while negative pixels correspond to the background region. Precision is determined by the ratio of true positive pixels predicted to the total number of positive pixels predicted. Sensitivity, or recall, is calculated as the ratio of true positive pixels predicted to the total number of positive pixels in the ground truth. The Jaccard index, or Intersection over Union (IoU), and the F1-score, or Dice similarity coefficient, are widely used evaluation metrics for comparing segmentation models. These metrics assess the overlap between positive predictions and ground truth while effectively penalizing false positives. Higher values approaching one indicate better performance in cell segmentation.

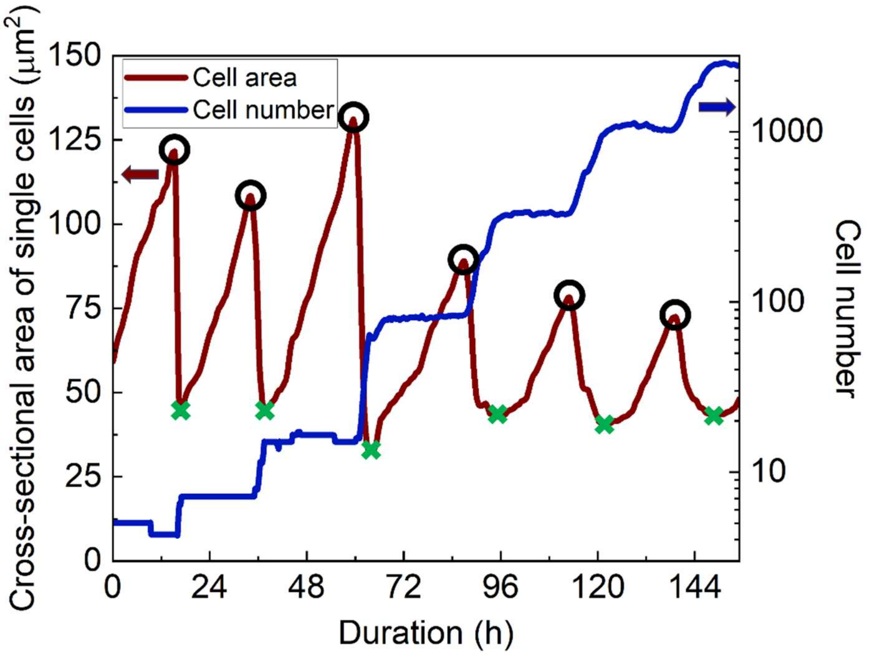

Fig. S4.

Inferring division dynamics from temporal cell size fluctuations. Peaks and valleys in the variation of average cell size within a single microcolony over the imaging duration (red line) were identified. Peaks in temporal cell size variation, indicated by black circles, correspond to the onset of cell division, while valleys, marked with green crosses, represent the sizes of newly divided daughter cells. The timing of these cell size fluctuations aligns with the step-like increase in cell number (blue line) within the monitored single microcolony.

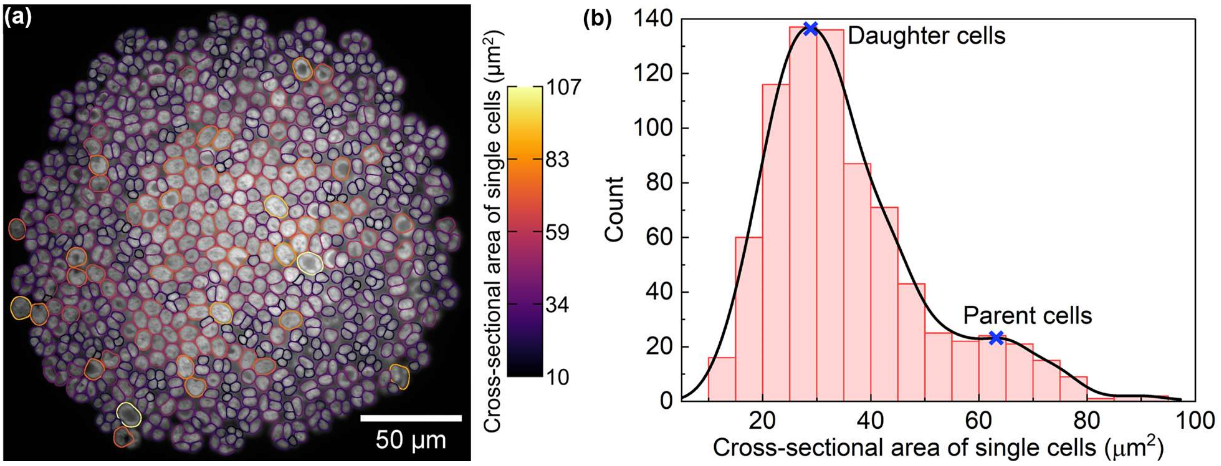

Fig. S5.

Decoupling the bimodal distribution of cell size at the onset of cell division episode. (a) Segmented cells within an isolated microcolony are displayed with an overlay of outlines, where the colours reflect the cross-sectional area of individual cells based on a colour map. (b) A histogram quantifies these cell sizes, revealing a bimodal distribution, with smaller cross-sectional areas corresponding to freshly born daughter cells and larger areas to undivided parent cells.

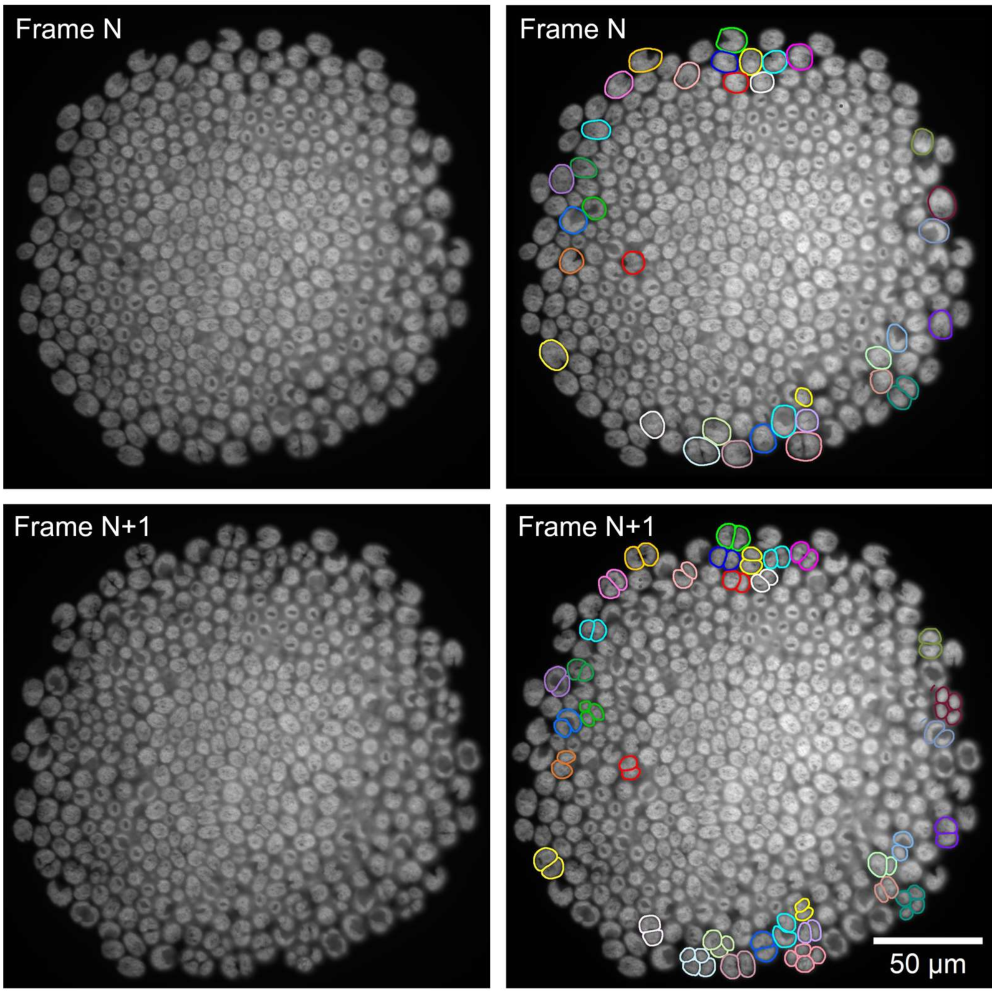

Fig. S6.

Spatiotemporal mapping of mitotic events across imaging frames. The positional correlation of cells entering the mitotic phase across consecutive frames can be analyzed, with the parent-daughter pairs colour-coded for identification on the right. Consecutive frames are separated by an imaging interval of one hour.

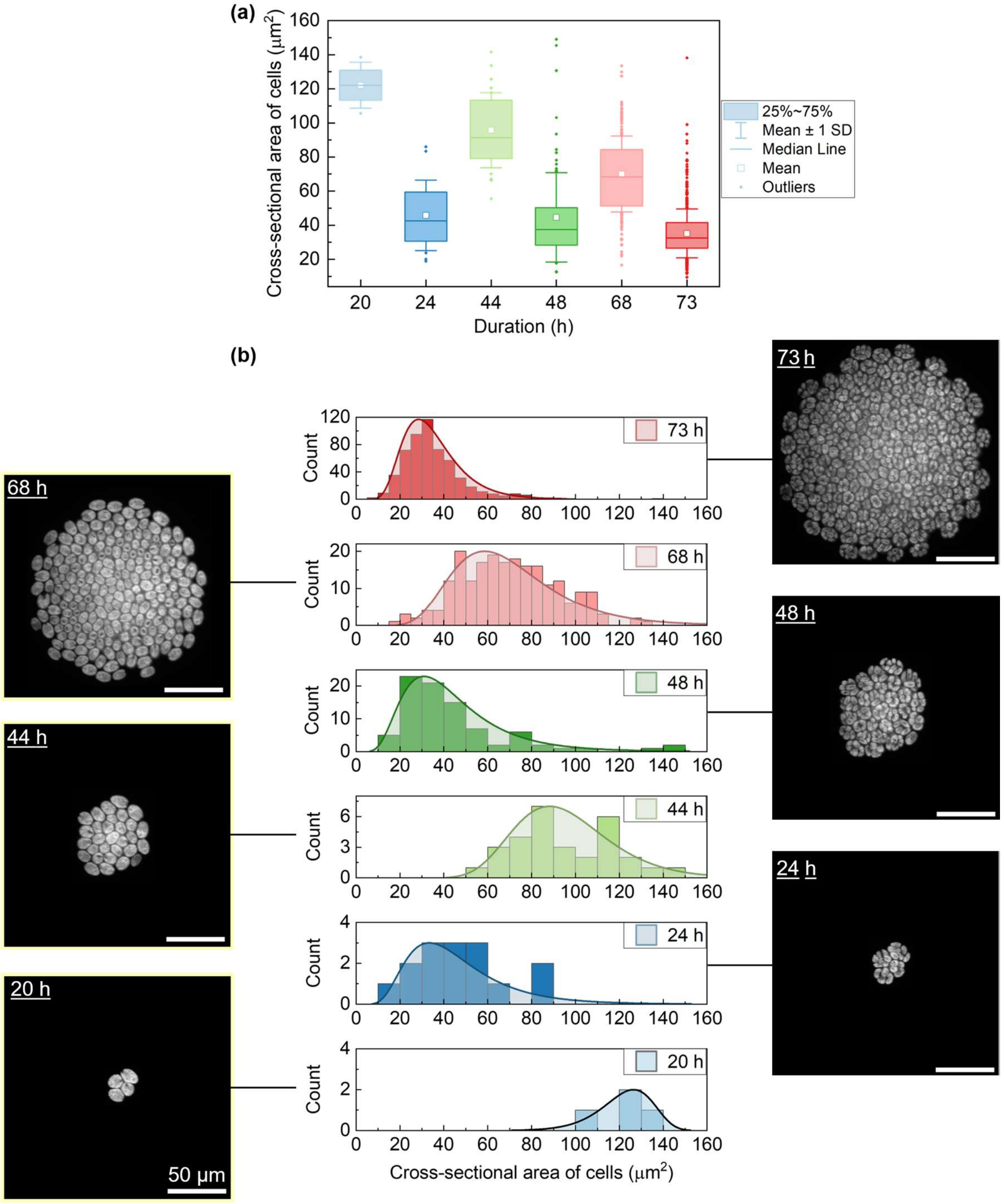

Fig. S7.

Cell size distributions as a function of time. (a) Aggregate quartile plots and (b) size distribution histograms of cell cross-sectional area at multiple time points before and after division episodes. Quartile plots summarize the temporal progression of cell size distributions, with medians, interquartile ranges, and outliers indicated. Histograms display the frequency distribution of cell sizes at each corresponding time point, illustrating population-level shifts in size before and after division events. Real fluorescent images of microcolonies at each time point are included to visually represent spatial organization and division progression.

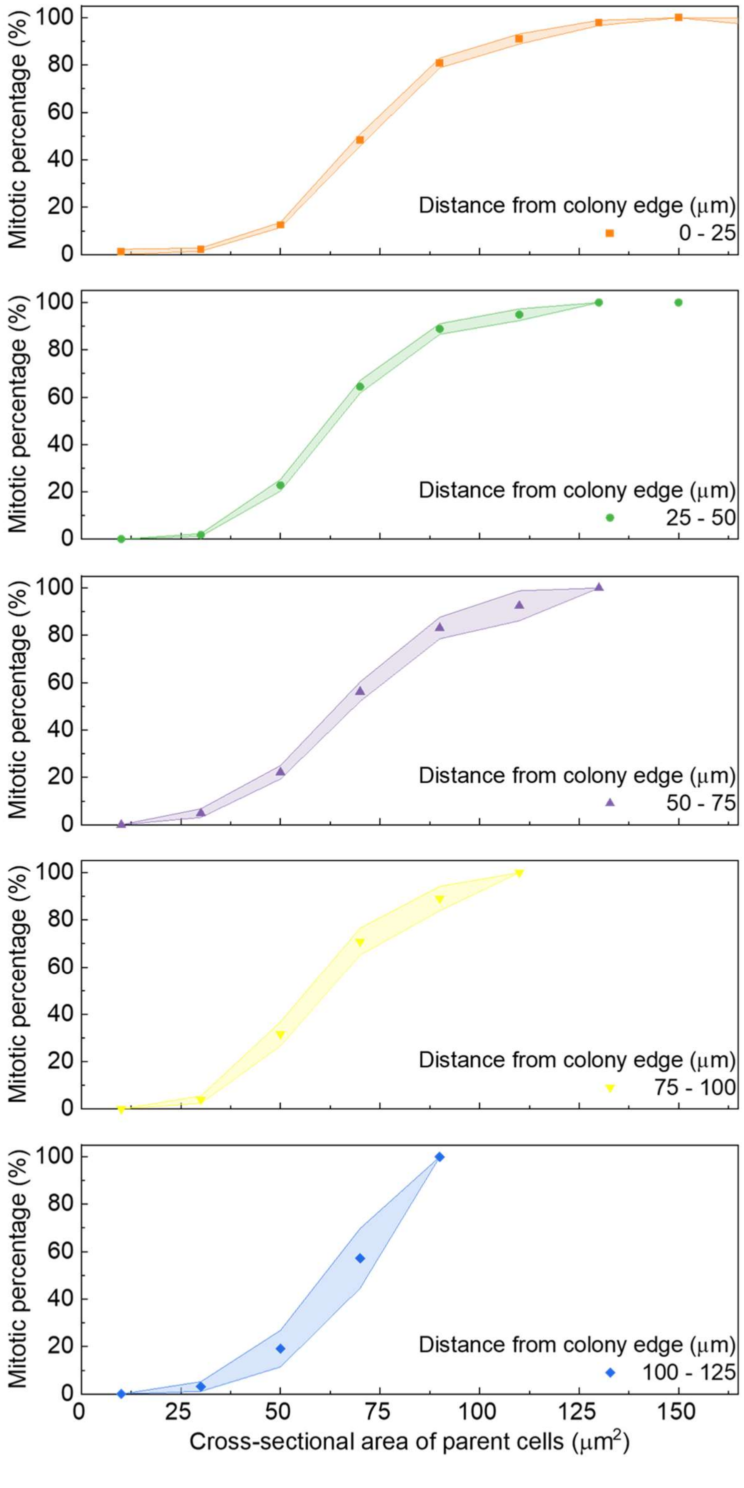

Fig. S8.

Stacked plots showing the relationship between mitotic percentage and cell size across different radial distances from the colony edge, measured under a 0.5% w/v agarose gel pad. Each panel corresponds to a specific radial segment within the microcolony. Shaded error bands represent the standard error of the mean (n = 21 microcolonies).

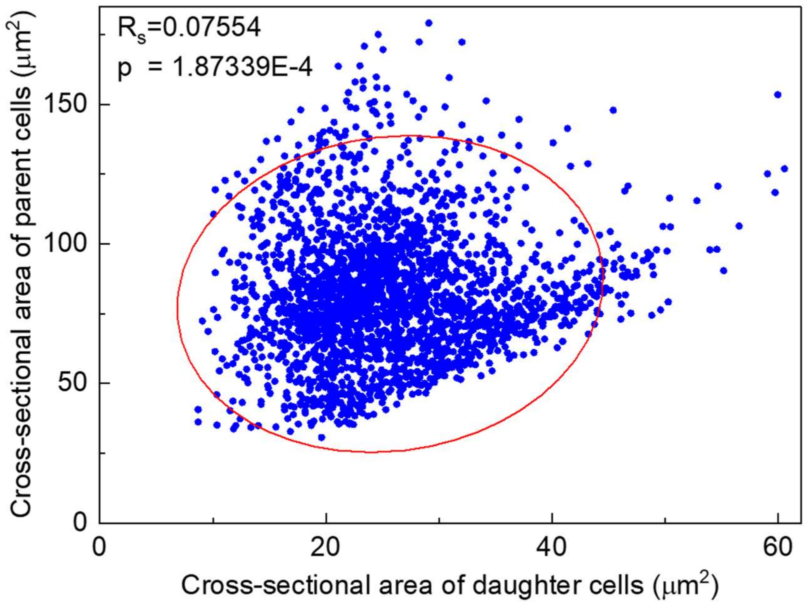

Fig. S9.

The relationship between parent and daughter cell sizes across a population of dividing cells. Each blue dot represents an individual division event, with parent cell size plotted on the y-axis and corresponding daughter cell size on the x-axis. The red ellipse indicates the 95% confidence region. The weak association between parent and daughter sizes is quantified by a Spearman's rank correlation coefficient (R_s) of 0.0755, indicating no significant correlation.

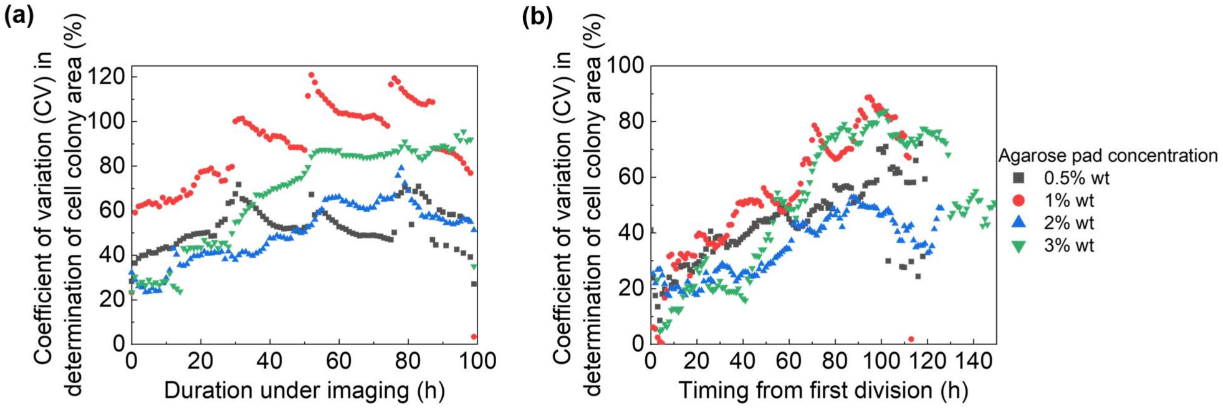

Fig. S10.

Coefficient of variation (CV) in colony area over time under varying agarose concentrations. The temporal evolution of the CV in cell colony area is shown for agarose gel pads of different concentrations: 0.5% wt (gray squares), 1% wt (red circles), 2% wt (blue upward triangles), and 3% wt (green downward triangles). Panel (a) shows the CV calculated with respect to absolute imaging time, starting from the first frame of acquisition, while panel (b) presents the CV relative to the timing of the first cell division event in each colony.

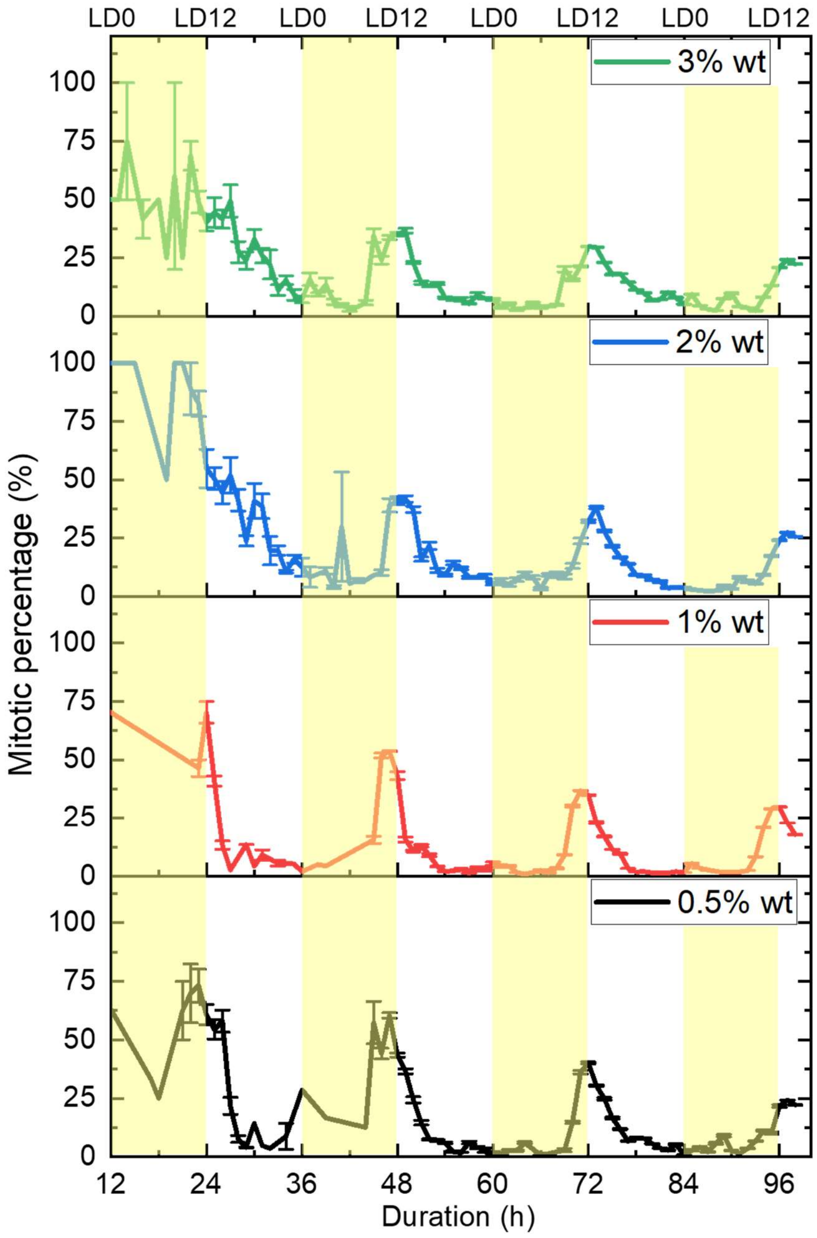

Fig. S11.

Stacked plots of mitotic percentage over time under varying agarose concentrations. Temporal profiles of mitotic percentage are shown for microcolonies grown under agarose gel pads of different concentrations (0.5%, 1%, 2%, and 3% wt), corresponding to the same data shown in Figure 4c of the main text. Each panel represents a different gel concentration, displaying the percentage of cells entering mitosis across the diurnal cycle (12h/12h). Yellow shading indicates light phases. Higher agarose concentrations resulted in delayed and reduced mitotic peaks compared to lower concentrations, demonstrating that increased gel stiffness affects both the timing and extent of mitotic entry during colony development. Error bars indicate standard error of mean (0.5% wt: n = 20; 1% wt: n = 10; 2% wt: n = 30; 3% wt: n = 30 microcolonies).

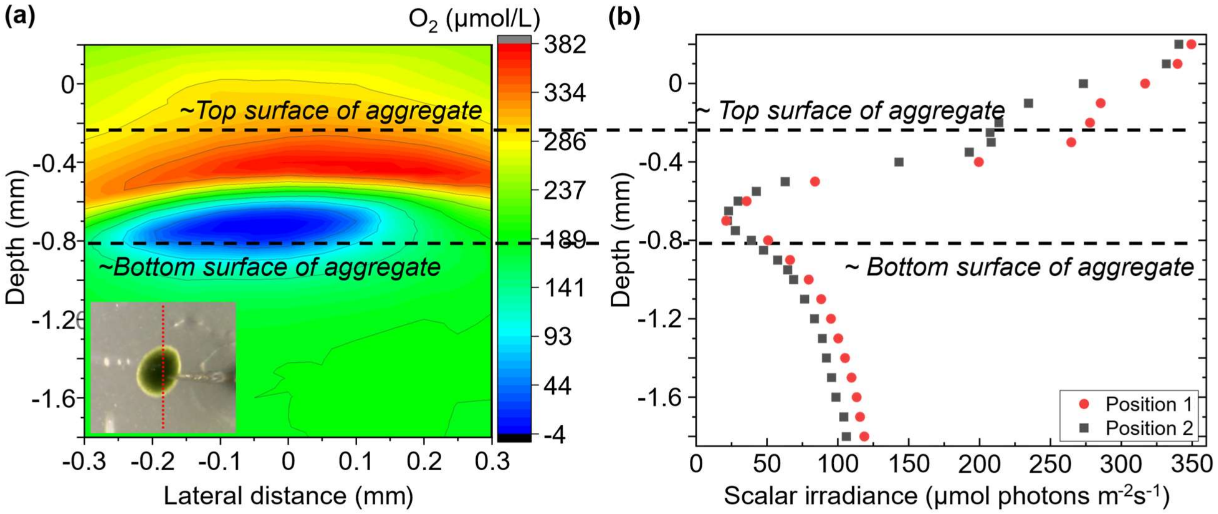

Fig. S12.

Mapping of O_2 concentration and light distribution around and across an algal aggregate. (a) Two-dimensional cross-sectional distribution of O_2 around and across an algal aggregate embedded in 1% wt agarose enriched with TAP medium, as interpolated from several microsensor measurements of concentration profiles under vertically incident illumination. The orientation of the cross-section is indicated by the white line in the inset photo (bottom left), which shows a top-down view of the same colony. The X-axis spans laterally across the colony center, while the Z-axis extends vertically through the agarose depth. (b) The corresponding profile of photon scalar irradiance (400-700 nm; µmol photons m^{-2} s^{-1}, as measured with a fiber-optic scalar irradiance microsensor. The dashed black lines indicate the depth corresponding to the top and bottom surface of the aggregate (shared reference position with panel a).

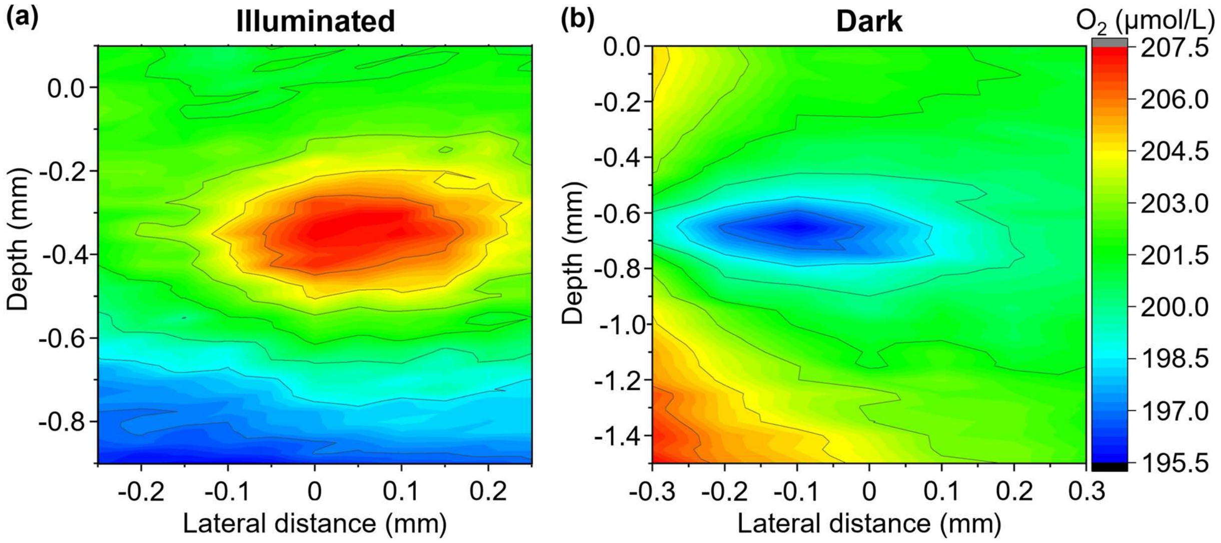

Fig. S13.

Mapping of O_2 concentration around and across microalgal aggregates under illuminated and dark conditions. (a) Two-dimensional oxygen distribution under steady illumination, measured along the cross-section of a single *C. reinhardtii* aggregate embedded in 1% wt agarose gel within Tris-minimal medium. The color gradient represents interpolated oxygen concentrations (μmol/L). (b) Oxygen profile measured under darkness in a separate, similarly sized aggregate.

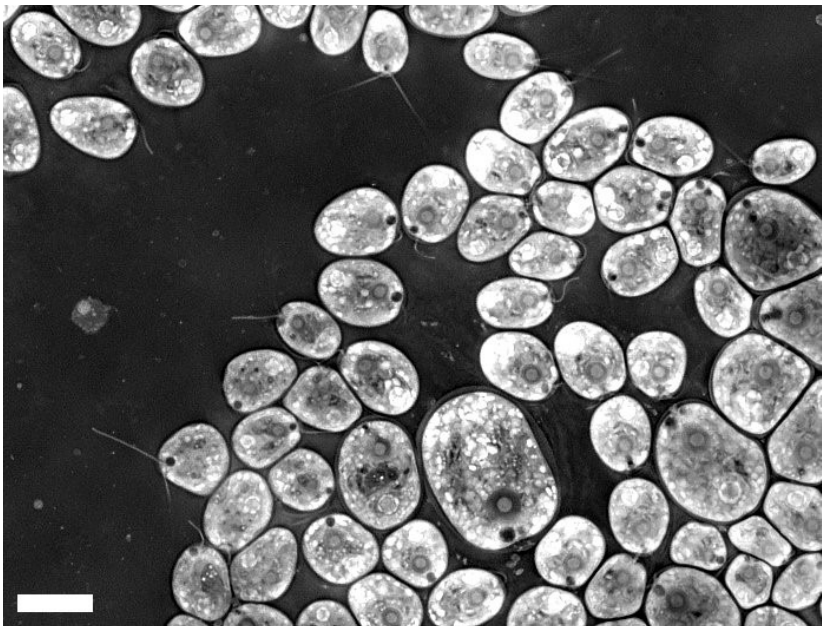

Fig. S14.
Holotomography of a gel-immobilised *C. reinhardtii* cell microcolony is presented with a colour scale representing the two-dimensional refractive index distribution in grayscale. This highlights the presence of flagella, which are visible around the microcolony periphery (scale bar = 5 µm).

Table S1. Fitted parameters for each distance group based on the modified threshold model. For each group, the optimized threshold size (S*), noise level (σ), and baseline division offset (Z_0) were independently fitted to the observed division data.

Distance from colony edge (μm)	S* (μm^3)	σ	Z_0
0-50	55.91	0.39	0.96
50-100	56.33	0.37	0.57
100-150	50.78	0.32	0.51

Table S2. Summary of parent cell size measurements (area in µm²) for different agarose gel concentrations and successive division numbers. The table reports the number of registered observations (Counts), mean cell area, standard deviation, and standard error of the mean, corresponding to Figure 3b.

Distance from colony edge (µm)	Number of successive divisions	Counts	Mean (µm^2)	Standard deviation (µm^2)	Standard error of mean (µm^2)
**0-50**	0	78	39.9	8.4	1.0
**0-50**	1	147	53.2	9.2	0.8
**0-50**	2	764	74.4	12.6	0.5
**0-50**	3	388	108.3	18.5	0.9
**0-50**	4	1	155.6	--	--
**50-100**	0	63	37.0	8.5	1.1
**50-100**	1	49	49.4	8.1	1.2
**50-100**	2	181	70.0	10.2	0.8
**50-100**	3	23	96.7	16.2	3.4
**100-150**	0	6	31.9	4.1	1.7
**100-150**	1	10	49.6	5.3	1.7
**100-150**	2	38	63.8	6.8	1.1

Table S3. Summary of parent cell size measurements (area in µm²) for different agarose gel concentrations and successive division numbers. The table reports the number of registered observations (Counts), mean cell area, standard deviation, and standard error of the mean, corresponding to Figure 4f.

Agarose gel concentration (% wt)	Number of successive divisions	Counts	Mean (µm^2)	Standard deviation (µm^2)	Standard error of mean (µm^2)
**0.5**	1	206	52.1	8.9	0.6
**3**	1	104	49.7	11.4	1.1
**0.5**	2	983	73.1	12.2	0.4
**3**	2	412	75.6	16.5	0.8
**0.5**	3	411	107.7	18.5	0.9
**3**	3	94	121.3	33.2	3.4
